# Charge-Carrier Mobility and Localization in Semiconducting Cu_2_AgBiI_6_ for Photovoltaic Applications

**DOI:** 10.1021/acsenergylett.1c00458

**Published:** 2021-04-07

**Authors:** Leonardo R. V. Buizza, Adam D. Wright, Giulia Longo, Harry C. Sansom, Chelsea Q. Xia, Matthew J. Rosseinsky, Michael B. Johnston, Henry J. Snaith, Laura M. Herz

**Affiliations:** † Department of Physics, University of Oxford, Clarendon Laboratory, Parks Road, Oxford, OX1 3PU, United Kingdom; ‡ Department of Mathematics, Physics and Electrical Engineering, University of Northumbria, Ellison Place, Newcastle-Upon-Tyne, NE1 8ST, United Kingdom; ¶ Department of Chemistry, University of Liverpool, Crown Street, Liverpool, L69 7ZD, United Kingdom

## Abstract

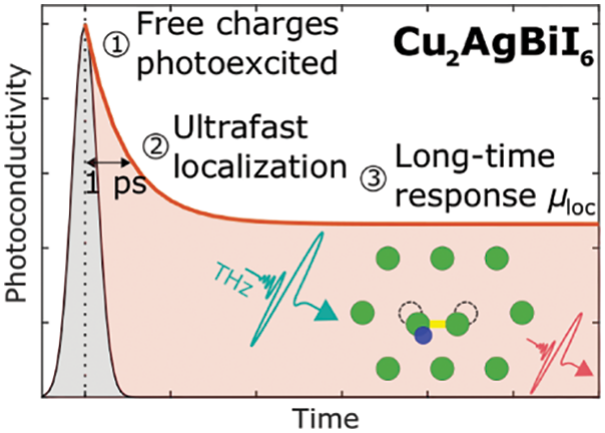

Lead-free silver–bismuth semiconductors have become increasingly popular materials for optoelectronic applications, building upon the success of lead halide perovskites. In these materials, charge-lattice couplings fundamentally determine charge transport, critically affecting device performance. In this study, we investigate the optoelectronic properties of the recently discovered lead-free semiconductor Cu_2_AgBiI_6_ using temperature-dependent photoluminescence, absorption, and optical-pump terahertz-probe spectroscopy. We report ultrafast charge-carrier localization effects, evident from sharp THz photoconductivity decays occurring within a few picoseconds after excitation and a rise in intensity with decreasing temperature of long-lived, highly Stokes-shifted photoluminescence. We conclude that charge carriers in Cu_2_AgBiI_6_ are subject to strong charge-lattice coupling. However, such small polarons still exhibit mobilities in excess of 1 cm^2^ V^–1^ s^–1^ at room temperature because of low energetic barriers to formation and transport. Together with a low exciton binding energy of ∼29 meV and a direct band gap near 2.1 eV, these findings highlight Cu_2_AgBiI_6_ as an attractive lead-free material for photovoltaic applications.

Developments in the field of lead-halide perovskites over the past decade have been rapid and impressive, notably with respect to new materials for solar cells.^1^ Power conversion efficiencies (PCEs) for single-junction cells have risen rapidly to over 25%, and perovskite-silicon tandems have surpassed 29%, indicating the potential for commercialization of perovskites for photovoltaic applications.^2,3^ However, concerns remain with regards to the toxicity of top-performing perovskite solar cell materials, most of which contain lead,^4^ as well as recurring difficulties around ensuring their long-term stability.^5^ The ideal solution would use light-harvesting materials that retain the excellent optoelectronic properties of lead-halide perovskites, notably tunable band gaps,^6^ high optical absorption,^1^ long charge-carrier lifetimes, and high charge-carrier mobilities,^7,8^ while removing lead from their composition. Such materials would eliminate concerns surrounding toxicity and promise high-efficiency, stable solar cells.^4,9^ Although replacing lead with tin has had some success, tin-based perovskite photovoltaics still struggle with substantially lower PCEs as well as higher instability and faster degradation, notably owing to the presence of tin vacancies which are easily formed.^10−13^

More recently, substantial interest has therefore emerged in so-called “double perovskites”,^4^ a vast class of over 90 000 materials, some of which can be lead-free.^14^ For such double perovskites, the divalent metal at the B-site of ABX_3_ single perovskites (such as Pb^2+^) is heterovalently swapped for a pair of cations with oxidation states 1+ and 3+, leading to a stoichiometry of A_2_BB’X_6_.^15,16^ The arrangement of atoms is very similar to that of standard ABX_3_ perovskites, with the 1+ and 3+ ions typically alternating between adjacent corner-sharing octahedral cages.^14,15^ The most frequently investigated material in this class is the all-inorganic double perovskite Cs_2_AgBiBr_6_, which is highly thermodynamically stable and has an indirect band gap with weak absorption onset around 2 eV as well as a strong direct gap near 3.1 eV.^15,17−20^ Spectroscopic and theoretical studies of this material have pointed toward low charge-carrier mobilities and long fundamental charge-carrier lifetimes,^18,20,21,23^ and work by Zelewski et al. suggested the existence of a color center as the source of photoluminescence in Cs_2_AgBiBr_6_,^19^ which would be indicative of strong electron–phonon coupling in this material. Relaxing the constraints of single- or double-perovskite structures allows for an even wider variety of materials to be explored for optoelectronic applications. These have ranged from all-inorganic materials such as quasi zero-dimensional tin halides Cs_4–*x*_A_*x*_Sn(Br_1–*y*_I_*y*_)_6_ (A = Rb^+^, K^+^),^24^ bismuth-based AgBiI_4_,^25^ CuBiI_4_,^26^ Cs_3_Bi_2_Br_9_,^27^ and Rb_4_Ag_2_BiBr_9_,^28^ the “hollow” tin perovskite {en}FASnI_3_ (en = ethylenediammonium),^29^ and (100) and (110) layered perovskites.^30−34^

The exploration of such wide material space has raised the question of how coupling between charge carriers and the lattice is affected, given this varies substantially as material structure is altered or as dimensionality is decreased, for example, by the creation of layered materials.^35−37^ The nature of electron–phonon coupling and charge localization plays a fundamental role in determining any intrinsic limits to the optoelectronic properties of materials and their suitability for photovoltaic applications. In the case of MAPbI_3_, coupling of charge carriers to longitudinal optical (LO) phonons limits charge-carrier mobilities to below 200 cm^2^V^–1^s^–1^: not as high as GaAs but sufficiently high to yield excellent solar cell devices given the very long charge-carrier lifetimes in MAPbI_3_.^38−41^ Larger couplings in other materials lead to strong charge-lattice interactions that have been described variously as a small polaron, as self-localization, self-trapping, or as the formation of a color center—phrases which often cover similar, or the same, couplings.^35,42−45^ There are extensive reviews of these effects in a variety of materials,^45−47^ and in this work we will use the terminology of a small polaron or self-trapped charge, where an initially photoexcited charge carrier rapidly relaxes into a state accompanied by a local lattice deformation.^48^ These interactions can be revealed by clear signals in spectroscopic studies investigating charge-carrier energetics, dynamics, and mobilities.^45,47,49^ Charge-carrier self-trapping has been reported across materials including all-inorganic KMgF_3_,^50^ in the bismuth-based systems Cs_2_AgBiBr_6_,^19,20,51^ Cs_3_Bi_2_Br_9_,^27^ and Rb_4_Ag_2_BiBr_9_,^28^ in layered perovskites,^30−34^ and in metal-halide materials such as PbBr_2_ and a number of alkali halides.^52−55^

As we have noted elsewhere,^47^ the strength of charge-carrier localization in a given material depends on its chemical composition, ease of structural distortion, and structural and electronic dimensionality. Further, it appears that in double perovskite or silver–bismuth materials, although the crystal structure can be three-dimensional, the electronic band structure is of lower dimensionality,^56^ making charge-carrier localization more likely.^19,20,51,57^ Studies across a variety of material classes demonstrate the general dependence of self-trapping on dimensionality: higher-dimensionality systems exhibit potential barriers between free and self-trapped states, meaning that self-trapping may occur only above certain temperatures and self-trapping rates display clear temperature dependence.^43,48,57^ In contrast, there appears to be little to no barrier for one- or zero-dimensional systems—a result that has been borne out both theoretically and experimentally.^35,58,59^ Given that charge-lattice interactions lower charge-carrier mobilities, the extent to which they prevail in any given semiconductor has an important bearing on photovoltaic performance. Because all charges interact with the surrounding lattice to some extent, an accurate assessment of the strength of such interactions and the resulting mobilities is therefore of central importance for any new light-harvesting material for photovoltaic applications.

In this work, we investigate the optoelectronic properties of a novel silver–bismuth-based semiconductor, the recently reported Cu_2_AgBiI_6_.^61^ An initial investigation of this material focused on its synthesis, structural, and compositional properties, stability, and basic optoelectronic properties.^61^ This early investigation revealed that Cu_2_AgBiI_6_ constitutes a heavily disordered network in which alternating layers of octahedral sites facilitated by the cubic close-packed iodide sublattice are partially occupied by Ag^+^ or Bi^3+^.^61^ This structure is shown in Figure 1a, with the unit cell enclosed by the black line, and the edge-sharing octahedral sites highlighted in purple (see Section 3 of the SI for a detailed analysis of XRD patterns and structure).

**Figure 1:**
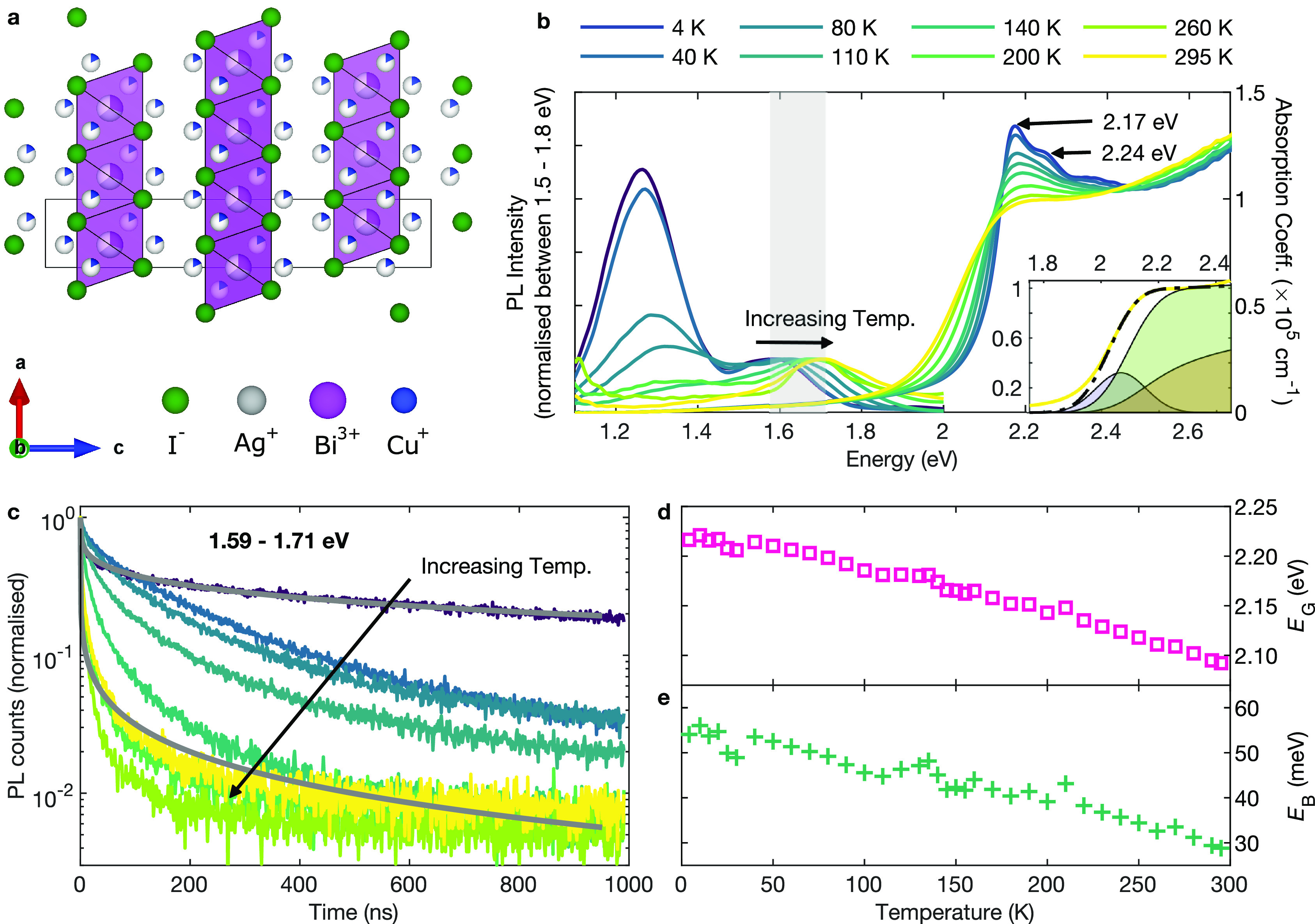
(a) Crystal structure of Cu_2_AgBiI_6_, with the edge-sharing octahedral layers highlighted in purple. The partial occupancy of the Ag^+^, Bi^3+^ and Cu^+^ sites is shown by the fractional filling of the circles at each ionic site. (b) Temperature-dependent photoluminescence and UV–visible absorption measurements of Cu_2_AgBiI_6_ thin films between 4–295 K. The PL peak blue-shifts with increasing temperature. The shaded region between 1.59–1.71 eV indicates the high-energy region from which TCSPC measurements were taken (shown in (c)) and from which peak counts were measured (shown in Figure S3 (c)). The inset shows the fit to the spectrum at 295 K using Elliott’s theory (black dashed line),^60^ with the shaded areas indicating the excitonic (blue) and continuum contributions without (brown) and with (green) Coulombic enhancement. See the Supporting Information for fits across more temperatures and the extracted broadening parameter Γ. (c) Time-resolved PL decays measured using TCSPC at a fluence of 200 nJ cm^–2^. The decays are very heterogeneous (nonexponential) at high temperatures, and become much longer-lived at low temperatures. The gray solid lines are fits to a stretched exponential at 4 and 295 K. See the Supporting Information for fits to all of the transients and extracted parameters. (d) Value of the band gap energy *E*_G_ extracted at each temperature using the Elliott fits. (e) Value of the exciton binding energy *E*_B_ extracted at each temperature using the Elliott fits.

An alternative description is that the octahedral network can be considered as a cation-disordered CdCl_2_ structure. The heavy cation disorder is apparent in the partial occupancy of each cation site, indicated by the fractional filling of the circles at each ionic site. The Cu^+^ partially occupies all of the tetrahedral sites facilitated by the cubic close-packed iodide sublattice. The structure shows a much higher level of cation disorder than the standard 3D corner-sharing octahedral networks present in (double) perovskites (i.e., it has a higher configurational entropy).^61^ For a more detailed outline of the structure, we direct readers to the original structural characterization by Sansom et al.^61^

The structure and chemical composition of Cu_2_AgBiI_6_ (including the presence of Ag^+^ and Bi^3+^, layered ordering and significant cation disorder) are likely to reduce the electronic dimensionality of the material^37^ and facilitate interactions of charge carriers with the lattice, making charge-carrier localization more likely to occur. Therefore, we here explore the nature of charge-carrier excited states in Cu_2_AgBiI_6_ based on temperature-dependent photoluminescence (PL), UV–visible absorption, time-correlated single-photon counting (TCSPC) and optical-pump terahertz-probe (OPTP) measurements. These experiments reveal several complementary pieces of evidence that together paint a picture of charge-carrier self-trapping and small-polaron formation in this material. The increase in intensity of long-lived, highly Stokes-shifted PL emission as temperatures are lowered, the discovery of an ultrafast decrease in photoconductivity that results in a long-lived localized charge-carrier population with lowered mobility, and the increasing mobility of this localized state with rising temperature all point toward a strong influence of the lattice on charge carriers in Cu_2_AgBiI_6_. We draw the conclusion that upon photoexcitation, self-trapped charge carriers or small polarons form rapidly within picoseconds in Cu_2_AgBiI_6_. However, we note that the temperature-activated mobility of such small polaron states still allows for sufficiently high charge-carrier mobilities at room temperature which, together with strong direct absorption near 2.1 eV and low exciton binding energy of 29 meV, make this material highly suitable for use in optoelectronic devices.

In order to establish the energetic distribution of electronic states in Cu_2_AgBiI_6_, we conducted temperature-dependent measurements of UV–visible absorption and photoluminescence spectra between 4–295 K (see Figure 1b). At 4 K, the absorption onset of Cu_2_AgBiI_6_ occurs at approximately 2 eV, which red-shifts to lower energies as the temperature is raised to 295 K. Further, the absorption spectrum of Cu_2_AgBiI_6_ at 4 K displays a clear excitonic feature with some fine structure, with two peaks visible at 2.17 and 2.24 eV (see Figure 1b). We analyzed the trends in absorption with temperature through fits to the spectra based on Elliott’s theory (see SI for details),^60^ accounting for both free electron and hole states and bound exciton contributions to the absorption, which are enhanced by Coulombic interactions, with bound excitonic contributions featuring prominently just below the band gap (see inset in Figure 1b and Figure S2).^62^ The resulting direct band gap energy *E*_G_ and exciton binding energy *E*_B_ for temperatures between 4–295 K are shown in panels d and e, respectively, of Figure 1. We note that the excellent fit of Elliott’s theory near the band edge and strong absorption coefficient values suggest a direct nature of this absorption onset.

Although the Elliott fits deviate slightly from the fine structure in the absorption spectra measured at low temperatures (see Figure S2), they are in good qualitative and quantitative agreement with the measured data. The fits confirm that the band gap shifts from 2.22 eV at 4 K down to 2.09 eV at 295 K, a trend that is opposite to that observed for conventional metal-halide perovskites such as MAPbI_3_, FAPbI_3_, or FASnI_3_,^12,39,63,64^ for which increasing temperatures lead to a blue-shift in band gap, owing to a combination of trends in electron–phonon coupling and the positive deformation potential of these materials.^65−67^ However, the temperature dependence of the band gap of Cu_2_AgBiI_6_ matches that observed for the well-studied silver–bismuth material Cs_2_AgBiBr_6_,^18,20^ and it is also similar to what is typically found in conventional inorganic semiconductors such as Si and GaAs, which obey Varshni’s rule of increasing band gaps with decreasing temperature.^68,69^

The exciton binding energy extracted from such Elliott fits falls from 54.1 to 28.8 meV between 4 K and room temperature. We note that a simpler determination of a binding energy by inspection of the fine structure observed near the absorption onset at 4 K, assuming that the adjacent peaks can be assigned to the *n* = 1, 2 Wannier excitonic states,^70^ gives *E*_B_ ∼ 80 meV. Such a low exciton binding energy of ∼29 meV at room temperature is exciting, given that excitonic features near the direct gap of the related semiconducting double perovskite Cs_2_AgBiBr_6_ are commensurate with much higher exciton binding energies nearer several hundred meV,^20,71^ and may partly derive from the lower direct band gap of Cu_2_AgBiI_6_ compared with Cs_2_AgBiBr_6_.^72^ We further note that the temperature-dependent values of the exciton binding energy we determine for Cu_2_AgBiI_6_ fall in between those measured for three-dimensional lead-halide perovskites,^62,64,73^ which are on the order of a few or a few tens of meVs, and those derived for two-dimensional Ruddlesden–Popper perovskites,^74^ of several hundred meVs. The layered structure of Cu_2_AgBiI_6_ likely supports some electronic confinement,^72^ leading to increased binding energies relative to three-dimensional metal-halide perovskites. However, this effect is somewhat mitigated by the fully three-dimensional iodide network, and unlike in layered Ruddlesden–Popper perovskites, there is no dielectric confinement effect that enhances exciton binding energies further.^74^ These factors, together with possible variations in charge-carrier effective masses, likely explain why the value of *E*_B_ found for Cu_2_AgBiI_6_ sits in between three-dimensional metal-halide perovskites and two-dimensional Ruddlesden–Popper perovskites. However, we note that the general trend of lower exciton binding energy with rising temperature is in line with estimates made using Elliot theory for both MAPbI_3_ and FAPbI_3_,^62,64^ where the exciton binding energy falls by a factor of 2–3 between 4–295 K. This reduction is mainly attributed to changes in the dielectric function with temperature, given the quadratic dependence of binding energy on dielectric permittivity for Wannier excitons.^62,64,73^ Overall, the room temperature exciton binding energy of ∼29 meV observed here for Cu_2_AgBiI_6_ compares with thermal energies (26 meV), meaning that the formation of uncorrelated electrons and holes should be highly effective, which will aid charge extraction in solar cell architectures.

The photoluminescence spectra of Cu_2_AgBiI_6_ (see Figure 1b) exhibit weak and fairly broad emission peaking at 1.71 eV at room temperature and display a large Stokes shift from the band gap of several hundred meV. As the temperature is lowered, this peak red-shifts slightly, down to 1.59 eV at 4 K, opposite to the trend observed for the absorption onset. We therefore consider it unlikely that this emission band is associated with radiative band-to-band recombination of free charge carriers. Instead, on the basis of our observations here and further investigations detailed below, we assign this emission band to a self-trapped state, for which a large Stokes shift and broad emission peak are highly characteristic.^48^ Studies of other silver–bismuth compounds, such as Cs_2_AgBiBr_6_^17,19^ and Rb_4_Ag_2_BiBr_9_,^28^ quasi-zero dimensional tin-halides,^24^ and layered perovskites,^30,32^ have similarly observed highly red-shifted emission from self-trapped states. Given the large cation disorder and edge-sharing octahedra in Cu_2_AgBiI_6_,^61^ we expect strong charge-lattice interactions to be present, resulting in highly Stokes-shifted emission, as has recently been suggested for a color center in Cs_2_AgBiBr_6_.^19^ In addition, we observe a narrowing of the emission spectra (see Figure 1b) and increase in intensity as the temperature is lowered (see Figure S3c), analogous to what has been observed for the emission attributed to a self-trapped state in both Rb_4_Ag_2_BiBr_9_^28^ and (EDBE)PbBr_4_ (EDBE = 2,2′-(ethylenedioxy)bis(ethylammonium)).^30^ As Figure S3c shows, the emission intensity around 1.59–1.71 eV in Cu_2_AgBiI_6_ increases by over a factor of 10 as the temperature is lowered, similar to previous observations of self-trapped states.^28,30,48^ However, we note that as the temperature is lowered, alternative nonradiative decay pathways mediated by lattice vibrations are significantly suppressed, which would lead to an enhancement of the emission from any radiative transition.

When the temperature is lowered below 200 K, a second emission peak emerges in the near-infrared around 1.35 eV, shifting down to 1.26 eV at 4 K and growing in intensity to surpass that of the higher-energy peak below 120 K (see Figure 1b and Figure S3a), and which could have multiple potential explanations. Given that this energy scale is very similar to the absorption at 1.24 eV observed through photothermal deflection spectroscopy (PDS) in the original investigation by Sansom et al.,^61^ we think it most likely arsises from a midgap defect state, similar to what is observed with Bi-doped CsAg_1–*x*_Na_*x*_InCl_6_ nanocrystals.^75^ Alternatively, the emission could be associated with an impurity, for example, the small quantities of Cu_2_BiI_5_ that were identified in the XRD measurements. We note that this impurity is unlikely to be BiI_3_ though, since this has been shown to absorb only weakly at the excitation wavelength near 400 nm and luminesce near 1.8 eV,^76−78^ where we do not find any significant intensity. Finally, an alternative explanation for the lower-energy emission band of Cu_2_AgBiI_6_ between 1.26–1.35 eV at low temperatures could be the disordered geometry of the structure supporting sites of distinctly different energies at different points in the lattice. Such effects have been proposed for Rb_4_Ag_2_BiBr_9_,^28^ for which self-trapped excitons on [BiBr_6_]^3−^ units give rise to three distinct peaks in the measured PL spectra that are separated by up to 240 meV.

To elucidate the dynamics relating to the charge–lattice interactions, we performed time-resolved spectroscopy on femtosecond through to microsecond time scales, using both nanosecond time-resolved PL and subpicosecond time-resolved THz photoconductivity techniques. We first report on time-resolved photoluminescence transients, measured using time-correlated single photon counting with ∼100 ps time resolution (Figure 1c). Such transients therefore allow for an examination of the time scales involved in the recombination of the higher-energy self-trapped emitting state. At high temperatures, the PL decays associated with the 1.59–1.71 eV emission band are very heterogeneous, exhibiting both very fast early components within the first few ns after excitation, as well as a tail with longer-lived dynamics. As the temperature is decreased, the initial fast component becomes much less dominant, and the longer-lived tail increases significantly in lifetime. Given the heterogeneity of these dynamics, stretched-exponential functions of the form *I* = *I*_0_*e*^–(*t*/τ_char_)^β^^ were fitted to these data in order to extract PL lifetimes,^79^ which are presented in Figure S3d of the SI. These fits yield an average PL lifetime of tens of nanoseconds at room temperature, rising to 4.1 μs at 4 K, for the high-energy PL band between 1.59–1.71 eV. Similar dynamics were measured for the low-energy emission, and these are shown in Figure S3b and discussed in the Supporting Information. Finally, time-resolved PL spectra were recorded at 4 K over the first 100 ns after excitation, which exhibit no energetic shifts over these time scales (see Figure S3e in the SI).

To interpret the observed PL dynamics, we note that self-trapped states have often been found to exhibit an increased lifetime as the temperature is lowered, as for example in zero-dimensional Cs_4_SnBr_6_^24^ and across the alkali halides,^80^ and by several orders of magnitude in the case of KBr, KI and RbI,^45,80^ similar to what we report here. However, trap-mediated recombination pathways may also be facilitated by lattice vibrations with increasing temperature, as seen in MAPbI_3_^63^ and mixed lead–tin FAPb_1–*x*_Sn_*x*_I_3_ perovskites,^12^ which may also partly explain the faster PL dynamics at higher temperatures. The observed heterogeneity of the PL decay from Cu_2_AgBiI_6_ (in particular at high temperature) is also in good agreement with the structural disorder deduced from XRD and the measured sizable Stokes shifts. However, the lack of shifts in the emission spectra over time evident in Figure S3e suggests that energetic relaxation either within electronic bands (e.g., from a direct to an indirect gap region),^70^ into the self-trapped state, or through charge migration to lattice locations with different emission energies, does not occur to a significant extent within the time window of 100 ps to 100 ns following excitation. Higher temporal resolution is therefore required in order to examine such effects.

In order to gain further insights into the dynamics of charge carriers and the potential formation of any self-trapped states, ultrafast optical-pump terahertz-probe spectroscopy was carried out using an amplified laser system (Spectra Physics, MaiTai – Empower – Spitfire) with a 400 nm pump pulse train (see Supporting Information for further experimental details). This technique provides insights into the fractional change in transmitted THz-field amplitude Δ*T*/*T*, which is proportional to the transient photoconductivity of a material, with subpicosecond time resolution (see Figure S6 in the SI), making it ideal for the study of charge-carrier localization effects. We note that 400 nm (3.1 eV) optical excitation was used in both the PL and OPTP measurements, well above the band gap, meaning that free carriers, rather than excitons, predominantly form immediately following photoexcitation in Cu_2_AgBiI_6_. Measurements at room temperature across several excitation fluences, shown in Figure 2a, reveal an ultrafast decay: the photoconductivity (proportional to the charge-carrier sum mobility μ and number of photoexcited charge-carriers *n*) decreases by a factor of 2 within a few ps, in a process that is fluence-independent (see Figure 2b inset). We note that the THz field probing Cu_2_AgBiI_6_ lies in the plane of the thin film: although there are clear 2D layers of octahedra in Cu_2_AgBiI_6_, the random lattice orientation across different grains in the thin film means that results from the OPTP measurements can be interpreted as a bulk average across all directions in the material.

**Figure 2:**
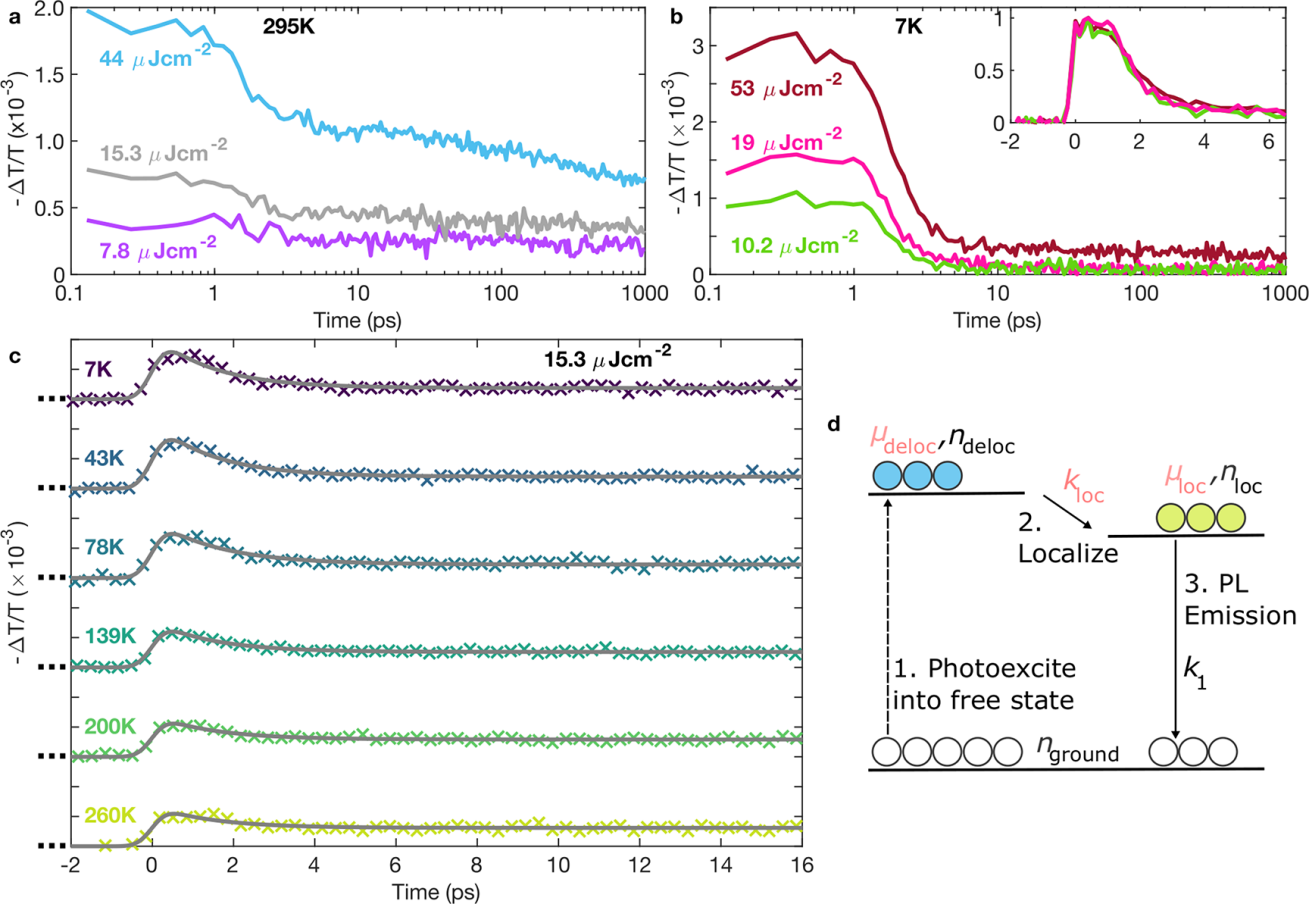
Fractional change in the transmitted THz-field amplitude for Cu_2_AgBiI_6_, proportional to the photoinduced THz conductivity, plotted as a function of time after excitation. Such OPTP transients are shown for a range of different excitation fluences for (a) 295 K and (b) 7 K. The inset in (b) shows the normalized traces at 7 K over the first 6 ps, indicating a lack of fluence dependence to the charge-carrier dynamics. (c) Early time temperature-dependent OPTP data measured at a fluence of 15.3 μJ cm^–2^ and fitted with a two-level mobility model, with the fits shown as gray solid lines. The two-level mobility model is explained schematically in (d), with fixed parameters shown in black and parameters that are fitted and extracted from the model in pink. The dotted line indicates the initial photoexcitation of charges, in our case due to pulsed laser excitation. See the main text and Supporting Information for further discussion of the model and parameter values extracted.

Previously reported time scales for the formation of self-trapped states in layered metal-halide perovskites are similar to those measured here, varying between hundreds of fs,^32,33,81^ through to tens of ps,^27^ and self-trapped exciton formation in other systems such as KI, NaCl, or Argon clusters has also been observed to occur on picosecond time scales.^82−84^ The lack of fluence dependence and presence of this ultrafast decay even at low excitation fluences (⩽ 10 μJ cm^–2^) rules out any bimolecular or Auger contributions, or exciton formation, as the origin of this component, once more indicating the likelihood of self-trapping processes leading to the observed ultrafast decay in charge-carrier conductivity.

To gain insights into the mechanisms limiting the charge-carrier mobility and to reveal any thermally activated processes, we conducted temperature-dependent THz photoconductivity (OPTP) measurements on Cu_2_AgBiI_6_ with the resulting transients shown in Figure 2b,c. Transient OPTP decays were measured out to 25 ps after excitation, and the temperature was varied between 7–290 K using a liquid-helium cryostat. Longer, fluence-dependent decays out to 1000 ps using logarithmically spaced points in time were also recorded at 7 K, and THz photoconductivity spectra were taken at several time delays for temperatures of 7 and 295 K (see Figure S4). The transient decays across all temperatures exhibit an initial fast decay component, followed by a long-lived decay. Through visual inspection and data fitting described in more detail below, we find the decay time of the initial ultrafast component to be temperature-invariant. However, at lower temperatures, the ultrafast component plays a more substantial role, that is, the contribution of the longer-lived component to the photoconductivity decreases as the temperature is reduced.

In order to verify the origin of the photoinduced conductivity response, we recorded spectra across the range of 0.4–2.5 THz at temperatures of 7 and 295 K and time delays of 0.2, 2, and 92 ps (see Figure S4d −i). The photoconductivity spectra exhibit very flat real and imaginary parts, with the latter being near-zero, indicative of Drude-like conductivity with short momentum scattering times.^85^ No sharp resonances were observed, suggesting the absence of discrete optically allowed transitions in the energy range of 2–10 meV (0.5–2.5 THz). These observations thus suggest that interexcitonic transitions do not fall into this range, in good agreement with the estimate of the binding energy obtained from the absorption measurements. Further, optical phonon modes are also absent in this energy region, and we therefore assume that the measured photoconductivity simply arises from photoinduced charge carriers and exhibits a linear dependence on charge-carrier mobility and population. We calculate a “Drude Factor”, following the method presented by Milot et al.,^63^ in order to assess the extent to which photoconductivity spectra follow a free-carrier response with short scattering times (zero imaginary part, constant real part). Our analysis yields values above 0.8 at all time delays for low (7 K) and room (295 K), indicating that Drude-like free-carrier conduction prevails (see Figure S4c) However, we note that at 7 K an interesting decrease in the Drude factor occurs over time, from 0.97 to 0.82 between 0.2 and 92 ps after excitation, which may be indicative of a stronger deviation from free-carrier like transport as charge carriers localize over time.

To assess the mobility of charge-carriers before and after the ultrafast charge-carrier localization, we fitted the early time transient decays in Figure 2c with a simple two-level model, shown schematically in Figure 2d and discussed in detail in the Supporting Information. The model assumes carriers are initially photoexcited high into the conduction band into a free-carrier state associated with a high mobility μ_deloc_ and a population *n*_deloc_, which determine the initial photoconductivity response at *t* = 0 ps. Following this, the mobility of charge carriers is reduced through the formation of a “localized” state, with mobility μ_loc_ and population *n*_loc_, with the localization rate given by *k*_loc_. From the localized state, charges will subsequently recombine down to the ground state over longer time scales, as observed in the time-resolved PL. We thus describe this recombination using long-time recombination rates *k*_1_, which we extract from the lifetimes obtained from the fits to the high-energy PL decays recorded with TSCPC at each temperature. While the initial free-carrier state could in principle also contribute photoluminescence through band-to-band recombination, no PL could actually be observed at energies close to the absorption onset, presumably because the free state depopulated too rapidly to contribute significantly and to be resolved in our TCSPC measurements. Both the free and the localized charge-carrier populations then contribute to the total recorded photoconductivity, given by Δ*σ* = *e* (*n*_deloc_μ_deloc_ + *n*_loc_μ_loc_). Through knowledge of the initial density of photons absorbed (*n*_0_ = 1. 59 × 10^18^ cm^–3^ — see Supporting Information for details), we may then extract a localization rate *k*_loc_ and effective charge-carrier mobilities μ_deloc_ and μ_loc_ at each temperature. Although this is a highly simplified schematic description of the charge-carrier dynamics (e.g., it does not account for the subsequent diffusion and recombination dynamics of charge carriers that, for example, give rise to the lower-energy emission around 1.25 eV), it allows us to focus on the key processes that dominate the charge-carrier dynamics over the first 25 ps: namely, charge-carrier localization from a high-mobility state into a low-mobility state.

In order to examine the charge transport mechanisms both before and after charge-carrier localization, we examine the respective mobility values extracted through the model and presented in Figure 3a. The value of μ_deloc_ describes the mobility of charge carriers that have been initially excited; these charges then can localize with a rate *k*_loc_ into a state with charge-carrier mobility μ_loc_. We find that the two species exhibit distinctly different temperature-dependencies in their mobilities; while μ_deloc_ decreases with increasing temperature, μ_loc_ is found to increase. To describe these trends, we fit a power-law dependence μ ∝*T*^*p*^ to both trends, yielding negative values of the exponent *p* = −0.33 ± 0.07 for μ_deloc_ and a positive *p* = 0.16 ± 0.05 for μ_loc_. Further, following Holstein and Emin,^43,44^ we fit an expression reflecting the thermally activated nature of the localized mobility, that is, , and obtain an activation energy of *E*_A_ = 0.4 ± 0.2 meV.

**Figure 3:**
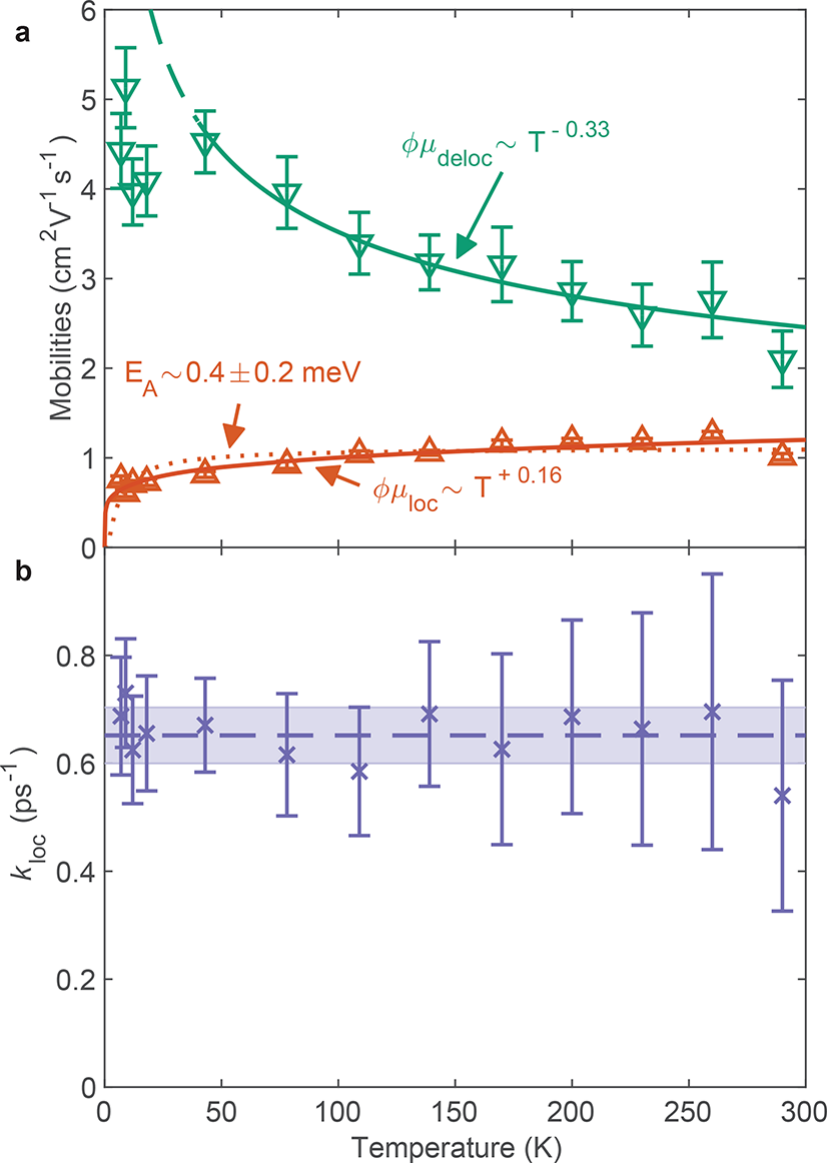
Temperature-dependence of (a) effective THz charge-carrier mobilities and (b) charge-carrier localization rates extracted from the two-level mobility model discussed in the main text. The values of μ_deloc_ for the lowest four temperatures were not included in the power-law fit, as this relation diverges unphysically as *T* → 0. The dashed line in (b) indicates the mean value of *k*_loc_ = 0.65 ± 0.05 ps^–1^ across all temperatures, showing the lack of temperature dependence of the self-trapping process.

In general, the temperature dependence of charge-carrier mobility depends strongly on the type of scattering or lattice coupling experienced by charge carriers, with the overall charge-carrier interactions potentially having several contributions at any given temperature.^49,72,86^ The negative temperature trend of the mobility of the free charge carriers is in accordance with relatively weak charge-lattice interactions (large polarons) dominating transport, while the positive trend for the localized state is a signature of a self-trapped or small-polaron state, as discussed further below. We note that the localization rate *k*_loc_ appears to be largely temperature independent (see Figure 3b) with an average value of *k*_loc_ = 0.65 ± 0.05 ps^–1^, indicating that the localization process itself is not temperature activated and therefore most likely barrier-free,^57^ suggesting that it may be caused by the low-dimensional electronic nature of Cu_2_AgBiI_6_ deriving from its structure and chemical composition.^35,37,87^ This would also be in accordance with recent reports of ultrafast self-trapping in Cs_2_AgBiBr_6_,^20,51^ which also has a zero-dimensional electronic structure,^37,87^ raising the question of the importance of the presence of silver and bismuth for charge-carrier localization to occur.^47^

In order for us to understand the temperature dependence of the mobility μ_deloc_ of free charge carriers in Cu_2_AgBiI_6_ prior to charge localization, that is, that of the initially formed large polarons, we contrast our findings against those made for the related hybrid metal-halide perovskites. The mobility of free charge carriers in metal-halide perovskites is governed by fundamentally similar mechanisms to those operating in classical inorganic semiconductors such as GaAs;^41^ however, some subtle differences may arise from metal-halide perovskites being mechanically soft and exhibiting temperature-dependent dielectric constants and anharmonic lattice vibrations.^65,86,88^ For the simple case of MAPbI_3_, Fröhlich coupling to longitudinal optical phonons has been shown to be the dominant mechanism affecting carrier mobilities, as expected for a polar semiconductor.^39,41^ However, there have been some discussions in the literature about the exact temperature dependence of the mobility expected for MAPbI_3_. While experiments mostly agree that mobilities vary with temperature with an exponent of *p* ≈ – 1.5 in the tetragonal phase,^41,63,89^ theoretical calculations have somewhat struggled to reproduce the observed trends conclusively. A simple application of Fröhlich’s theory incorporating experimental parameters yielded a *p* = −0.46 dependency,^90^ while closer attention to the way in which couplings were distributed across the many longitudinal optical modes in MAPbI_3_ yielded much more negative values of *p*,^86^ closer to experiment, and even more negative values in the low-temperature orthorhombic phase where acoustic phonon scattering begins to dominate. In addition, recent microscopic models accounting for lattice fluctuations have suggested that anharmonicity in MAPbI_3_ may give rise to negative exponents near *p* = −2.^91^ We also note that other metal-halide perovskites, including FAPbI_3_,^64^ FASnI_3_,^13^ and Cs_2_AgBiBr_6_,^22^ have exhibited mobility exponents different from *p* ≈ – 1.5, implying a variety of charge-lattice interactions and scattering mechanisms that need to be taken into account across different materials. The low value of *p* = −0.33 measured here for Cu_2_AgBiI_6_ may therefore derive from the presence of multiple scattering mechanisms, some of which may relate to intrinsic electron–phonon coupling, while others derive from extrinsic effects correlating with the partially layered ordering in the structure and high cation disorder of this material.^61^ High charge-carrier mobilities are crucial for photovoltaic applications,^92^ and the values of μ_deloc_ = 2.1–5.1 cm^2^ V^–1^ s^–1^ presented here are a promising initial measurement. Further, the flatter temperature dependence of the delocalized charge-carrier mobility, relative to conventional metal-halide perovskites,^41,63^ suggests that extrinsic factors, such as poor crystallinity, still play a significant role, meaning that suitable improvements in processing protocols may still improve charge-carrier mobilities substantially.^41^

Following the initial localization step, charge carriers exhibit a positive temperature dependence of increasing μ_loc_ with increasing temperature. Such behavior is highly indicative of temperature-activated “hopping” transport typical of localized charge carriers,.^43,44^ Theoretical studies of such small polarons in crystal lattices have predicted a positive temperature dependence of the carrier mobility,^43,58^ confirmed by experiments across material classes such as chalcogenide glasses or perovskite-oxides,^44,93,94^ while contributions from polarons and energetic disorder to charge transport in organic semiconductors have been studied extensively, with mobilities of charges or neutral excitons in some organic semiconductors also rising with increasing temperature.^95,96^ The formation of a self-trapped state or small polaron agrees well with the measurement of positive temperature dependence for μ_loc_, and the very low value for the activation energy of 0.4 ± 0.2 meV is in excellent agreement with the invariance of the localization rate *k*_loc_ with temperature.

The measurement of positive temperature dependence for μ_loc_ in Cu_2_AgBiI_6_ thus supports the notion that a self-trapped state or small polaron forms soon after photoexcitation. The fast localization time scales (see Figure 3b) that have little temperature dependence imply a small barrier to the formation of a self-trapped state,^57,59^ as observed in lower-dimensional perovskites such as (*N*–MEDA)[PbBr_4_]^32^ (*N*–MEDA = N1-methylethane-1,2-diammonium), and are in accordance with the structure of Cu_2_AgBiI_6_, with edge-sharing octahedra and octahedral layering. Thus, the values measured for μ_loc_ are likely to result from a combination of hopping transport of a self-trapped state, as well as any remaining free-carrier contribution, for example from either free electrons or holes that do not undergo self-trapping, as we cannot be certain at this point which carrier types are subject to self-trapping. As discussed in a recent review article,^47^ this reduction in charge-carrier mobility can be qualitatively interpreted as a reduction in charge-carrier scattering time or an increase in charge-carrier effective mass. There may be slight changes in carrier scattering times when charge carriers localize in Cu_2_AgBiI_6_, as quantified by changes in the calculated “Drude Factor” above, but these are likely to be quite small and would not lead to substantial changes in charge-carrier mobility. Instead, given that we observe charge carriers localizing into small polarons which then carry a local lattice deformation with them as they “hop” between lattice sites, we argue that this localization translates to a substantial increase in carrier effective mass, which leads to the observed reduction in charge-carrier mobility.

Overall, photoexcitation therefore initially creates a number of free electrons and holes forming large polarons, which contribute to the initial photoconductivity with an overall electron–hole sum mobility given by μ_deloc_. These free charges rapidly localize on a picosecond time scale into a self-trapped or small polaron state, reducing the measured photoconductivity, with the remaining signal comprising contributions from remaining free charges and hopping transport of the localized state, described overall as μ_loc_. The variation in charge-carrier mobility of both localized and free states with temperature then gives rise to the variations in observed OPTP transients shown in Figure 2c. As a guide for the suitability of Cu_2_AgBiI_6_ for optoelectronic devices, we use the measured values for μ_loc_ and *k*_1_ at room temperature to calculate an estimate of the charge-carrier diffusion length  as ∼400 nm. This value is similar to the typical layer thickness implemented in many thin-film photovoltaic and optoelectronic devices,^1^ suggesting that this material has good promise for use in these architectures. In addition, further improvements are likely achievable through optimization of materials processing, defect passivation and device-based optimization, as has been the case for conventional metal-halide perovskites.

We note that in principle, the presence of an initial high-mobility electronic state and a longer-time low-mobility state could alternatively derive from the relaxation of charges from a direct band into an indirect band. Studies of other bismuth-halide based materials such as Cs_2_AgBiBr_6_, Cs_2_AgBiCl_6_, and Rb_4_Ag_2_BiBr_9_ have revealed indirect band gaps,^16,28^ with higher-lying direct gaps contributing more prominently to absorption. This has been shown to be a general property of silver–bismuth double perovskites for which both Ag and Bi orbitals contribute to the band structure at the valence band minimum or conduction band maximum.^97^ Further, theoretical calculations of the band structure of silver–bismuth Cs_2_AgBiX_6_ (X = Br, Cl) show that the direct bands at the Γ point are more strongly curved than the lower-lying indirect bands at the L and X points.^16^ Given that higher curvature implies lower charge-carrier effective masses and thus higher charge-carrier mobilities, charge-carrier relaxation from the direct gap to the indirect gap would lead to lower mobilities and thus decreased photoconductivity. Although, as explained above, Cu_2_AgBiI_6_ is not a double perovskite, it could have similar contributions to the band structure, making it plausible that our measured OPTP transients are caused by ultrafast relaxation of charge carriers into an indirect gap following initial excitation across the direct gap. However, this explanation cannot easily support the observed switch in temperature-dependent behavior of the charge-carrier mobility between the delocalized and localized states, as it is unlikely for charge-carrier scattering mechanisms to change so profoundly simply because of relaxation into an indirect gap. Indeed, charge-carrier mobilities in Si and GaP, which are indirect semiconductors with a higher-lying direct gap, decrease as temperature increases because of increased phonon scattering—the opposite of what is observed here.^98−101^ If charge carriers in Cu_2_AgBiI_6_ relaxed into an indirect band within a few ps, we would then expect μ_loc_ to display a similar temperature dependence to what is observed for μ_deloc_.

A similar argument is valid to exclude charge-carrier cooling as the potential source of the early time reduction in photoconductivity: given that higher-lying bands in metal-halide perovskites have lower curvature (higher effective mass), charge-carrier relaxation into the more curved (lower effective mass) band extrema should lead to a rise in charge-carrier mobility and thus photoconductivity. This has been observed in excess-energy dependent OPTP spectroscopy in conventional metal-halide perovskites^102,103^ but is the opposite to what is observed here for Cu_2_AgBiI_6_. Given this, we argue that the thermally activated hopping mobility μ_loc_ indicates the formation of small polarons or self-trapped carriers in Cu_2_AgBiI_6_, as outlined above, at the lowest point of the bandstructure.^43,44,48^

In conclusion, we have presented clear evidence for strong charge-lattice coupling leading to self-trapping processes in the recently discovered semiconductor Cu_2_AgBiI_6_. Self-trapping of charge carriers is apparent in the temperature-dependent PL and leads to highly red-shifted emission with large Stokes shifts. The self-trapping process is also prominent in the charge-carrier dynamics: at low temperatures the photoluminescence exhibits a very long lifetime, on the order of microseconds, while there is an ultrafast decay in photoconductivity that is visible at all temperatures. Localized charge carriers have lower mobilities, leading to decreased photoconductivity on a picosecond time scale. The lack of temperature dependence of the localization rate and very low activation energy obtained for the “hopping” transport imply a low energetic barrier to self-trapping, which may derive from the lowered electronic dimensionality of this material. These findings will thus allow connections to be made between order, electronic, and lattice structure design and the extent to which such localization tends to occur. Overall, we note that while the presence of a self-trapped state and ultrafast localization somewhat lowers charge-carrier transport, this effect is not substantial at room temperature, partly because small-polaron motion is temperature-activated. At room temperature, this process therefore only reduces the measured photoconductivity by a factor of 2, still leaving sufficiently high values near 1 cm^2^ V^–1^ s^–1^ to enable efficient photovoltaic devices. Together with an attractive bandgap near 2.1 eV for large-gap subcells in multilayer photovoltaic architectures or visible light emission, and low exciton binding energies comparable to thermal energies at room temperature, Cu_2_AgBiI_6_ therefore presents an attractive new semiconductor for optoelectronic applications.

## Supporting Information Available

Experimental details, X-ray diffraction data, details of the Elliott fits to the absorption spectra, characterisations of the low-energy photoluminescence, time-resolved photoconductivity spectra, and an explanation of the two-level mobility model used to fit the temperature-dependent OPTP transients (PDF)

## References

[ref1] Green M. A.; Ho-Baillie A.; Snaith H. J. The emergence of perovskite solar cells. Nat. Photonics 2014, 8, 506–514. 10.1038/nphoton.2014.134.

[ref2] Green M. A.; Dunlop E. D.; Levi D. H.; Hohl-Ebinger J.; Yoshita M.; Ho-Baillie A. W. Solar cell efficiency tables (version 54). Prog. Photovoltaics 2019, 27, 565–575. 10.1002/pip.3171.

[ref3] National Renewable Energy Laboratory (NREL). Best Research-Cell Efficiency Chart. https://www.nrel.gov/pv/cell-efficiency.html (accessed Feb. 20, 2021).

[ref4] Giustino F.; Snaith H. J. Toward Lead-Free Perovskite Solar Cells. ACS Energy Letters 2016, 1, 1233–1240. 10.1021/acsenergylett.6b00499.

[ref5] Boyd C. C.; Cheacharoen R.; Leijtens T.; McGehee M. D. Understanding Degradation Mechanisms and Improving Stability of Perovskite Photovoltaics. Chem. Rev. 2019, 119, 3418–3451. 10.1021/acs.chemrev.8b00336. 30444609

[ref6] Protesescu L.; Yakunin S.; Bodnarchuk M. I.; Krieg F.; Caputo R.; Hendon C. H.; Yang R. X.; Walsh A.; Kovalenko M. V. Nanocrystals of Cesium Lead Halide Perovskites (CsPbX_3_, X = Cl, Br, and I): Novel Optoelectronic Materials Showing Bright Emission with Wide Color Gamut. Nano Lett. 2015, 15, 3692–3696. 10.1021/nl5048779. 25633588 PMC4462997

[ref7] Wehrenfennig C.; Eperon G. E.; Johnston M. B.; Snaith H. J.; Herz L. M. High Charge Carrier Mobilities and Lifetimes in Organolead Trihalide Perovskites. Adv. Mater. 2014, 26, 1584–1589. 10.1002/adma.201305172. 24757716 PMC4722848

[ref8] Stranks S. D.; Eperon G. E.; Grancini G.; Menelaou C.; Alcocer M. J. P.; Leijtens T.; Herz L. M.; Petrozza A.; Snaith H. J. Electron-hole diffusion lengths exceeding 1 micrometer in an organometal trihalide perovskite absorber. Science 2013, 342, 341–344. 10.1126/science.1243982. 24136964

[ref9] Flora G.; Gupta D.; Tiwari A. Toxicity of lead: A review with recent updates. Interdiscip. Toxicol. 2012, 5, 47–58. 10.2478/v10102-012-0009-2. 23118587 PMC3485653

[ref10] Stoumpos C. C.; Malliakas C. D.; Kanatzidis M. G. Semiconducting tin and lead iodide perovskites with organic cations: Phase transitions, high mobilities, and near-infrared photoluminescent properties. Inorg. Chem. 2013, 52, 9019–9038. 10.1021/ic401215x. 23834108

[ref11] Noel N. K.; Stranks S. D.; Abate A.; Wehrenfennig C.; Guarnera S.; Haghighirad A.-A.; Sadhanala A.; Eperon G. E.; Pathak S. K.; Johnston M. B.; Petrozza A.; Herz L. M.; Snaith H. J. Lead-free organic-inorganic tin halide perovskites for photovoltaic applications. Energy Environ. Sci. 2014, 7, 3061–3068. 10.1039/C4EE01076K.

[ref12] Parrott E. S.; Green T.; Milot R. L.; Johnston M. B.; Snaith H. J.; Herz L. M. Interplay of structural and optoelectronic properties in formamidinium mixed tin-lead triiodide perovskites. Adv. Funct. Mater. 2018, 28, 1802803 10.1002/adfm.201802803.

[ref13] Milot R. L.; Klug M. T.; Davies C. L.; Wang Z.; Kraus H.; Snaith H. J.; Johnston M. B.; Herz L. M. The effects of doping density and temperature on the optoelectronic properties of formamidinium tin triiodide thin films. Adv. Mater. 2018, 30, 1804506 10.1002/adma.201804506. 30222220

[ref14] Filip M. R.; Giustino F. The geometric blueprint of perovskites. Proc. Natl. Acad. Sci. U. S. A. 2018, 115, 5397–5402. 10.1073/pnas.1719179115. 29735683 PMC6003477

[ref15] Volonakis G.; Filip M. R.; Haghighirad A. A.; Sakai N.; Wenger B.; Snaith H. J.; Giustino F. Lead-free halide double perovskites via heterovalent substitution of noble metals. J. Phys. Chem. Lett. 2016, 7, 1254–1259. 10.1021/acs.jpclett.6b00376. 26982118

[ref16] Filip M. R.; Hillman S.; Haghighirad A. A.; Snaith H. J.; Giustino F. Band gaps of the lead-free halide double perovskites Cs_2_BiAgCl_6_ and Cs_2_BiAgBr_6_ from theory and experiment. J. Phys. Chem. Lett. 2016, 7, 2579–2585. 10.1021/acs.jpclett.6b01041. 27322413

[ref17] Slavney A. H.; Hu T.; Lindenberg A. M.; Karunadasa H. I. A Bismuth-Halide Double Perovskite with Long Carrier Recombination Lifetime for Photovoltaic Applications. J. Am. Chem. Soc. 2016, 138, 2138–2141. 10.1021/jacs.5b13294. 26853379

[ref18] Schade L.; Wright A. D.; Johnson R. D.; Dollmann M.; Wenger B.; Nayak P. K.; Prabhakaran D.; Herz L. M.; Nicholas R.; Snaith H. J.; Radaelli P. G. Structural and optical properties of Cs_2_AgBiBr_6_ double perovskite. ACS Energy Letters 2019, 4, 299–305. 10.1021/acsenergylett.8b02090.

[ref19] Zelewski S. J.; Urban J. M.; Surrente A.; Maude D. K.; Kuc A.; Schade L.; Johnson R. D.; Dollmann M.; Nayak P. K.; Snaith H. J.; Radaelli P.; Kudrawiec R.; Nicholas R. J.; Plochocka P.; Baranowski M. Revealing the nature of photoluminescence emission in the metal-halide double perovskite Cs_2_AgBiBr_6_. J. Mater. Chem. C 2019, 7, 8350–8356. 10.1039/C9TC02402F.

[ref20] Wright A. D.; Buizza L. R. V.; Savill K. J.; Longo G.; Snaith H. J.; Johnston M. B.; Herz L. M. Ultrafast Excited-State Localization in Cs_2_AgBiBr_6_ Double Perovskite. J. Phys. Chem. Lett. 2021, 12, 3352–3360. 10.1021/acs.jpclett.1c00653. 33783218 PMC8154850

[ref21] Bartesaghi D.; Slavney A. H.; Gélvez-Rueda M. C.; Connor B. A.; Grozema F. C.; Karunadasa H. I.; Savenije T. J. Charge carrier dynamics in Cs_2_AgBiBr_6_ double perovskite. J. Phys. Chem. C 2018, 122, 4809–4816. 10.1021/acs.jpcc.8b00572. PMC5846080 29545908

[ref22] Hutter E. M.; Gélvez-Rueda M. C.; Bartesaghi D.; Grozema F. C.; Savenije T. J. Band-like charge transport in Cs_2_AgBiBr_6_ and mixed antimony–bismuth Cs_2_AgBi_1–x_Sb_x_Br_6_ halide double perovskites. ACS Omega 2018, 3, 11655–11662. 10.1021/acsomega.8b01705. 30288465 PMC6166227

[ref23] Hoye R. L. Z.; Eyre L.; Wei F.; Brivio F.; Sadhanala A.; Sun S.; Li W.; Zhang K. H. L.; MacManus-Driscoll J. L.; Bristowe P. D.; Friend R. H.; Cheetham A. K.; Deschler F. Fundamental carrier lifetime exceeding 1 μs in Cs_2_AgBiBr_6_ double perovskite. Adv. Mater. Interfaces 2018, 5, 1800464 10.1002/admi.201800464.

[ref24] Benin B. M.; Dirin D. N.; Morad V.; Wörle M.; Yakunin S.; Rainó G.; Nazarenko O.; Fischer M.; Infante I.; Kovalenko M. V. Highly emissive self-trapped excitons in fully inorganic zero-dimensional tin halides. Angew. Chem., Int. Ed. 2018, 57, 11329–11333. 10.1002/anie.201806452. PMC6175341 29999575

[ref25] Sansom H. C.; Whitehead G. F.; Dyer M. S.; Zanella M.; Manning T. D.; Pitcher M. J.; Whittles T. J.; Dhanak V. R.; Alaria J.; Claridge J. B.; Rosseinsky M. J. AgBiI_4_ as a Lead-Free Solar Absorber with Potential Application in Photovoltaics. Chem. Mater. 2017, 29, 1538–1549. 10.1021/acs.chemmater.6b04135.

[ref26] Hu Z.; Wang Z.; Kapil G.; Ma T.; Iikubo S.; Minemoto T.; Yoshino K.; Toyoda T.; Shen Q.; Hayase S. Solution-processed air-stable copper bismuth iodide for photovoltaics. ChemSusChem 2018, 11, 2930–2935. 10.1002/cssc.201800815. 29920992

[ref27] Liu C.; Wang Y.; Geng H.; Zhu T.; Ertekin E.; Gosztola D.; Yang S.; Huang J.; Yang B.; Han K.; Canton S. E.; Kong Q.; Zheng K.; Zhang X. Asynchronous photoexcited electronic and structural relaxation in lead-free perovskites. J. Am. Chem. Soc. 2019, 141, 13074–13080. 10.1021/jacs.9b04557. 31361482

[ref28] Sharma M.; Yangui A.; Whiteside V. R.; Sellers I. R.; Han D.; Chen S.; Du M.-H.; Saparov B. Rb_4_Ag_2_BiBr_9_: A lead-free bisible light absorbing halide semiconductor with improved stability. Inorg. Chem. 2019, 58, 4446–4455. 10.1021/acs.inorgchem.8b03623. 30767513

[ref29] Ke W.; Stoumpos C. C.; Zhu M.; Mao L.; Spanopoulos I.; Liu J.; Kontsevoi O. Y.; Chen M.; Sarma D.; Zhang Y.; Wasielewski M. R.; Kanatzidis M. G. Enhanced photovoltaic performance and stability with a new type of hollow 3D perovskite {en}FASnI_3_. Science Advances 2017, 3, e1701293 10.1126/sciadv.1701293. 28875173 PMC5576879

[ref30] Dohner E. R.; Jaffe A.; Bradshaw L. R.; Karunadasa H. I. Intrinsic white-light emission from layered hybrid perovskites. J. Am. Chem. Soc. 2014, 136, 13154–13157. 10.1021/ja507086b. 25162937

[ref31] Dohner E. R.; Hoke E. T.; Karunadasa H. I. Self-assembly of broadband white-light emitters. J. Am. Chem. Soc. 2014, 136, 1718–1721. 10.1021/ja411045r. 24422494

[ref32] Hu T.; Smith M. D.; Dohner E. R.; Sher M.-J.; Wu X.; Trinh M. T.; Fisher A.; Corbett J.; Zhu X.-Y.; Karunadasa H. I.; Lindenberg A. M. Mechanism for broadband white-light emission from two-dimensional (110) hybrid perovskites. J. Phys. Chem. Lett. 2016, 7, 2258–2263. 10.1021/acs.jpclett.6b00793. 27246299

[ref33] Cortecchia D.; Yin J.; Bruno A.; Lo S.-Z. A.; Gurzadyan G. G.; Mhaisalkar S.; Brédas J.-L.; Soci C. Polaron self-localization in white-light emitting hybrid perovskites. J. Mater. Chem. C 2017, 5, 2771–2780. 10.1039/C7TC00366H.

[ref34] Yangui A.; Garrot D.; Lauret J. S.; Lusson A.; Bouchez G.; Deleporte E.; Pillet S.; Bendeif E. E.; Castro M.; Triki S.; Abid Y.; Boukheddaden K. Optical investigation of broadband white-light emission in self-assembled organic-inorganic perovskite (C_6_H_11_NH_3_)_2_PbBr_4_. J. Phys. Chem. C 2015, 119, 23638–23647. 10.1021/acs.jpcc.5b06211.

[ref35] Kabanov V. V.; Mashtakov O. Y. Electron localization with and without barrier formation. Phys. Rev. B: Condens. Matter Mater. Phys. 1993, 47, 6060–6064. 10.1103/PhysRevB.47.6060. 10004555

[ref36] Connor B. A.; Leppert L.; Smith M. D.; Neaton J. B.; Karunadasa H. I. Layered halide double perovskites: Dimensional reduction of Cs_2_AgBiBr_6_. J. Am. Chem. Soc. 2018, 140, 5235–5240. 10.1021/jacs.8b01543. 29575889

[ref37] Xiao Z.; Meng W.; Wang J.; Mitzi D. B.; Yan Y. Searching for promising new perovskite-based photovoltaic absorbers: the importance of electronic dimensionality. Mater. Horiz. 2017, 4, 206–216. 10.1039/C6MH00519E.

[ref38] Wehrenfennig C.; Liu M.; Snaith H. J.; Johnston M. B.; Herz L. M. Charge-carrier dynamics in vapour-deposited films of the organolead halide perovskite CH_3_NH_3_PbI_3–x_Cl_x_. Energy Environ. Sci. 2014, 7, 2269–2275. 10.1039/C4EE01358A.

[ref39] Wright A. D.; Verdi C.; Milot R. L.; Eperon G. E.; Pérez-Osorio M. A.; Snaith H. J.; Giustino F.; Johnston M. B.; Herz L. M. Electron-phonon coupling in hybrid lead halide perovskites. Nat. Commun. 2016, 7, 11755 10.1038/ncomms11755. PMC4894981 27225329

[ref40] Sendner M.; Nayak P. K.; Egger D. A.; Beck S.; Müller C.; Epding B.; Kowalsky W.; Kronik L.; Snaith H. J.; Pucci A.; Lovrinčić R. Optical phonons in methylammonium lead halide perovskites and implications for charge transport. Mater. Horiz. 2016, 3, 613–620. 10.1039/C6MH00275G.

[ref41] Herz L. M. Charge-carrier mobilities in metal halide perovskites: fundamental mechanisms and limits. ACS Energy Letters 2017, 2, 1539–1548. 10.1021/acsenergylett.7b00276.

[ref42] Landau L. D. On the motion of electrons in a crystal lattice. Physikalische Zeitschrift der Sowjetunion 1933, 3, 644–645.

[ref43] Holstein T. Studies of polaron motion: Part II. The “small” polaron. Ann. Phys. 1959, 8, 343–389. 10.1016/0003-4916(59)90003-X.

[ref44] Emin D.; Seager C. H.; Quinn R. K. Small-polaron hopping motion in some chalcogenide glasses. Phys. Rev. Lett. 1972, 28, 813–816. 10.1103/PhysRevLett.28.813.

[ref45] Williams R.; Song K. The self-trapped exciton. J. Phys. Chem. Solids 1990, 51, 679–716. 10.1016/0022-3697(90)90144-5.

[ref46] Stoneham A. M.; Gavartin J.; Shluger A. L.; Kimmel A. V.; Mũoz Ramo D.; Rønnow H. M.; Aeppli G.; Renner C. Trapping, self-trapping and the polaron family. J. Phys.: Condens. Matter 2007, 19, 255208 10.1088/0953-8984/19/25/255208.

[ref47] Buizza L. R. V.; Herz L. M. Polarons and charge localisation in metal-halide semiconductors for photovoltaic and light-emitting devices. Adv. Mater. 2021, 33, 2007057 10.1002/adma.202007057. 33955594

[ref48] Song K. S.; Williams R. T. Self-Trapped Excitons; Springer: Berlin Heidelberg, 1993; p 404.

[ref49] Herz L. M. How lattice dynamics moderate the electronic properties of metal-halide perovskites. J. Phys. Chem. Lett. 2018, 9, 6853–6863. 10.1021/acs.jpclett.8b02811. 30422667

[ref50] Lewis J. T.; Kolopus J. L.; Sonder E.; Abraham M. M. Reorientation and motion of the self-trapped hole in KMgF_3_. Phys. Rev. B 1973, 7, 810–818. 10.1103/PhysRevB.7.810.

[ref51] Wu B.; Ning W.; Xu Q.; Manjappa M.; Feng M.; Ye S.; Fu J.; Lie S.; Yin T.; Wang F.; et al. Strong self-trapping by deformation potential limits photovoltaic performance in bismuth double perovskite. Science Advances 2021, 7, eabd3160 10.1126/sciadv.abd3160. 33597239 PMC7888938

[ref52] Iwanaga M.; Azuma J.; Shirai M.; Tanaka K.; Hayashi T. Phys. Rev. B: Condens. Matter Mater. Phys. 2002, 65, 214306 10.1103/PhysRevB.65.214306.

[ref53] Iwanaga M.; Hayashi T. Exciton-relaxation dynamics in lead halides. J. Lumin. 2003, 102–103, 663–668. 10.1016/S0022-2313(02)00619-1.

[ref54] Unuma Y.; Masumoto Y.; Shionoya S.; Nishimura H. Dynamical aspects of self-trapping of 1s Excitons in RbI and KI. J. Phys. Soc. Jpn. 1983, 52, 4277–4282. 10.1143/JPSJ.52.4277.

[ref55] Castner T. G.; Känzig W. The electronic structure of V-centers. J. Phys. Chem. Solids 1957, 3, 178–195. 10.1016/0022-3697(57)90023-9.

[ref56] Xiao Z.; Song Z.; Yan Y. From Lead Halide Perovskites to Lead-Free Metal Halide Perovskites and Perovskite Derivatives. Adv. Mater. 2019, 31, 1803792 10.1002/adma.201803792. 30680809

[ref57] Emin D.; Holstein T. Adiabatic Theory of an Electron in a Deformable Continuum. Phys. Rev. Lett. 1976, 36, 323–326. 10.1103/PhysRevLett.36.323.

[ref58] Emin D. Lattice relaxation and small-polaron hopping motion. Phys. Rev. B 1971, 4, 3639–3651. 10.1103/PhysRevB.4.3639.

[ref59] Morrissey F. X.; Mance J. G.; Van Pelt A. D.; Dexheimer S. L. Femtosecond dynamics of exciton localization: self-trapping from the small to the large polaron limit. J. Phys.: Condens. Matter 2013, 25, 144204 10.1088/0953-8984/25/14/144204. 23478998

[ref60] Elliott R. J. Intensity of optical absorption by excitons. Phys. Rev. 1957, 108, 1384 10.1103/PhysRev.108.1384.

[ref61] Sansom H. C. Highly Absorbing Lead-Free Semiconductor Cu_2_AgBiI_6_ for Photovoltaic Applications from the Quaternary CuI-AgI-BiI_3_ Phase Space. J. Am. Chem. Soc. 2021, 143, 3983–3992. 10.1021/jacs.1c00495. 33684283 PMC8041282

[ref62] Davies C. L.; Filip M. R.; Patel J. B.; Crothers T. W.; Verdi C.; Wright A. D.; Milot R. L.; Giustino F.; Johnston M. B.; Herz L. M. Bimolecular recombination in methylammonium lead triiodide perovskite is an inverse absorption process. Nat. Commun. 2018, 9, 293 10.1038/s41467-017-02670-2. 29348550 PMC5773627

[ref63] Milot R. L.; Eperon G. E.; Snaith H. J.; Johnston M. B.; Herz L. M. Temperature-dependent charge-carrier dynamics in CH_3_NH_3_PbI_3_ perovskite thin films. Adv. Funct. Mater. 2015, 25, 6218–6227. 10.1002/adfm.201502340.

[ref64] Davies C. L.; Borchert J.; Xia C. Q.; Milot R. L.; Kraus H.; Johnston M. B.; Herz L. M. Impact of the organic cation on the optoelectronic properties of formamidinium lead triiodide. J. Phys. Chem. Lett. 2018, 9, 4502–4511. 10.1021/acs.jpclett.8b01628. 30036475

[ref65] Whalley L. D.; Skelton J. M.; Frost J. M.; Walsh A. Phonon anharmonicity, lifetimes, and thermal transport in CH_3_NH_3_PbI_3_ from many-body perturbation theory. Phys. Rev. B: Condens. Matter Mater. Phys. 2016, 94, 220301 10.1103/PhysRevB.94.220301.

[ref66] Saidi W. A.; Kachmar A. Effects of electron-phonon coupling on electronic properties of methylammonium lead iodide perovskites. J. Phys. Chem. Lett. 2018, 9, 7090–7097. 10.1021/acs.jpclett.8b03164. 30514084

[ref67] Saidi W. A.; Poncé S.; Monserrat B. Temperature dependence of the energy levels of methylammonium lead iodide perovskite from first-principles. J. Phys. Chem. Lett. 2016, 7, 5247–5252. 10.1021/acs.jpclett.6b02560. 27973908

[ref68] Varshni Y. P. Temperature dependence of the energy gap in semiconductors. Physica 1967, 34, 149–154. 10.1016/0031-8914(67)90062-6.

[ref69] D’Innocenzo V.; Grancini G.; Alcocer M. J. P.; Kandada A. R. S.; Stranks S. D.; Lee M. M.; Lanzani G.; Snaith H. J.; Petrozza A. Excitons versus free charges in organo-lead tri-halide perovskites. Nat. Commun. 2014, 5, 3586 10.1038/ncomms4586. 24710005

[ref70] Pelant I.; Valenta J. Luminescence Spectroscopy of Semiconductors; Oxford University Press, 2012.

[ref71] Kentsch R.; Scholz M.; Horn J.; Schlettwein D.; Oum K.; Lenzer T. Exciton Dynamics and Electron-Phonon Coupling Affect the Photovoltaic Performance of the Cs_2_AgBiBr_6_ Double Perovskite. J. Phys. Chem. C 2018, 122, 25940–25947. 10.1021/acs.jpcc.8b09911.

[ref72] Yu P. Y.; Cardona M. Fundamentals of semiconductors: physics and materials properties, 4th ed.; Springer: Heidelberg Dordrecht London New York, 2010.

[ref73] Herz L. M. Charge-Carrier Dynamics in Organic-Inorganic Metal Halide Perovskites. Annu. Rev. Phys. Chem. 2016, 67, 65–89. 10.1146/annurev-physchem-040215-112222. 26980309

[ref74] Hong X.; Ishihara T.; Nurmikko A. V. Dielectric confinement effect on excitons in PbI 4 -based layered semiconductors. Phys. Rev. B: Condens. Matter Mater. Phys. 1992, 45, 6961–6964. 10.1103/PhysRevB.45.6961. 10000465

[ref75] Locardi F.; Sartori E.; Buha J.; Zito J.; Prato M.; Pinchetti V.; Zaffalon M. L.; Ferretti M.; Brovelli S.; Infante I.; De Trizio L.; Manna L. Emissive Bi-Doped Double Perovskite Cs_2_Ag_1–x_Na_x_InCl_6_ Nanocrystals. ACS Energy Letters 2019, 4, 1976–1982. 10.1021/acsenergylett.9b01274.

[ref76] Brandt R. E.; Kurchin R. C.; Hoye R. L. Z.; Poindexter J. R.; Wilson M. W. B.; Sulekar S.; Lenahan F.; Yen P. X. T.; Stevanović V.; Nino J. C.; Bawendi M. G.; Buonassisi T. Investigation of bismuth triiodide (BiI_3_) for photovoltaic applications. J. Phys. Chem. Lett. 2015, 6, 4297–4302. 10.1021/acs.jpclett.5b02022. 26538045

[ref77] Williamson B. W.; Eickemeyer F. T.; Hillhouse H. W. Solution-processed BiI_3_ films with 1.1 eV quasi-Fermi level splitting: the role of water, temperature, and solvent during processing. ACS Omega 2018, 3, 12713–12721. 10.1021/acsomega.8b00813. 31457997 PMC6644407

[ref78] Hamdeh U. H.; Nelson R. D.; Ryan B. J.; Bhattacharjee U.; Petrich J. W.; Panthani M. G. Solution-processed BiI_3_ thin films for photovoltaic applications: improved carrier collection via solvent annealing. Chem. Mater. 2016, 28, 6567–6574. 10.1021/acs.chemmater.6b02347.

[ref79] Johnston D. C. Stretched exponential relaxation arising from a continuous sum of exponential decays. Phys. Rev. B: Condens. Matter Mater. Phys. 2006, 74, 184430 10.1103/PhysRevB.74.184430.

[ref80] Fischbach J. U.; Fröhlich D.; Kabler M. N. Recombination luminescence lifetimes and the self-trapped excition in alkali halides. J. Lumin. 1973, 6, 29–43. 10.1016/0022-2313(73)90092-6.

[ref81] Zhang R.; Mao X.; Yang Y.; Yang S.; Zhao W.; Wumaier T.; Wei D.; Deng W.; Han K. Air-stable, lead-free zero-dimensional mixed bismuth-antimony perovskite single crystals with ultra-broadband emission. Angew. Chem., Int. Ed. 2019, 58, 2725–2729. 10.1002/anie.201812865. 30663267

[ref82] Hirai M.; Suzuki Y.; Hattori H.; Ehara T.; Kitamura E. Picosecond laserphotolysis on photo-induced defect formation process in KI crystal. J. Phys. Soc. Jpn. 1987, 56, 2948–2963. 10.1143/JPSJ.56.2948.

[ref83] Tokizaki T.; Makimura T.; Akiyama H.; Nakamura A.; Tanimura K.; Itoh N. Femtosecond cascade-excitation spectroscopy for nonradiative deexcitation and lattice relaxation of the self-trapped exciton in NaCl. Phys. Rev. Lett. 1991, 67, 2701–2704. 10.1103/PhysRevLett.67.2701. 10044495

[ref84] Lietard A.; Piani G.; Briant M.; Gaveau M.-A.; Faisan S.; Mazet V.; Soep B.; Mestdagh J.-M.; Poisson L. Self-trapping relaxation decay investigated by time-resolved photoelectron spectroscopy. Phys. Chem. Chem. Phys. 2018, 20, 11206–11214. 10.1039/C7CP06789E. 29632903

[ref85] Ulatowski A. M.; Herz L. M.; Johnston M. B. Terahertz Conductivity Analysis for Highly Doped Thin-Film Semiconductors. J. Infrared, Millimeter, Terahertz Waves 2020, 41, 1431–1449. 10.1007/s10762-020-00739-6.

[ref86] Poncé S.; Schlipf M.; Giustino F. Origin of low carrier mobilities in halide perovskites. ACS Energy Letters 2019, 4, 456–463. 10.1021/acsenergylett.8b02346.

[ref87] Zhao X.-G.; Yang D.; Ren J.-C.; Sun Y.; Xiao Z.; Zhang L. Rational Design of Halide Double Perovskites for Optoelectronic Applications. Joule 2018, 2, 1662–1673. 10.1016/j.joule.2018.06.017.

[ref88] Onoda-Yamamuro N.; Matsuo T.; Suga H. Dielectric study of CH_3_NH_3_PbX_3_ (X = Cl, Br, I). J. Phys. Chem. Solids 1992, 53, 935–939. 10.1016/0022-3697(92)90121-S.

[ref89] Savenije T. J.; Ponseca C. S.; Kunneman L.; Abdellah M.; Zheng K.; Tian Y.; Zhu Q.; Canton S. E.; Scheblykin I. G.; Pullerits T.; Yartsev A.; Sundström V. Thermally activated exciton dissociation and recombination control the carrier dynamics in organometal halide perovskite. J. Phys. Chem. Lett. 2014, 5, 2189–2194. 10.1021/jz500858a. 26279532

[ref90] Frost J. M. Calculating polaron mobility in halide perovskites. Phys. Rev. B: Condens. Matter Mater. Phys. 2017, 96, 195202 10.1103/PhysRevB.96.195202.

[ref91] Mayers M. Z.; Tan L. Z.; Egger D. A.; Rappe A. M.; Reichman D. R. How Lattice and Charge Fluctuations Control Carrier Dynamics in Halide Perovskites. Nano Lett. 2018, 18, 8041–8046. 10.1021/acs.nanolett.8b04276. 30387614

[ref92] Johnston M. B.; Herz L. M. Hybrid perovskites for photovoltaics: charge-carrier recombination, diffusion, and radiative efficiencies. Acc. Chem. Res. 2016, 49, 146–154. 10.1021/acs.accounts.5b00411. 26653572

[ref93] Iguchi E.; Ueda K.; Jung W. H. Conduction in LaCoO_3_ by small-polaron hopping below room temperature. Phys. Rev. B: Condens. Matter Mater. Phys. 1996, 54, 17431–17437. 10.1103/PhysRevB.54.17431. 9985867

[ref94] Hulea I. N.; Fratini S.; Xie H.; Mulder C. L.; Iossad N. N.; Rastelli G.; Ciuchi S.; Morpurgo A. F. Tunable Fröhlich polarons in organic single-crystal transistors. Nat. Mater. 2006, 5, 982–986. 10.1038/nmat1774. 17086169

[ref95] Fishchuk I. I.; Kadashchuk A.; Hoffmann S. T.; Athanasopoulos S.; Genoe J.; Bässler H.; Köhler A. Unified description for hopping transport in organic semiconductors including both energetic disorder and polaronic contributions. Phys. Rev. B: Condens. Matter Mater. Phys. 2013, 88, 125202 10.1103/PhysRevB.88.125202.

[ref96] Coropceanu V.; Cornil J.; da Silva Filho D. A.; Olivier Y.; Silbey R.; Brédas J.-L. Charge transport in organic semiconductors. Chem. Rev. 2007, 107, 926–952. 10.1021/cr050140x. 17378615

[ref97] Slavney A. H.; Connor B. A.; Leppert L.; Karunadasa H. I. A pencil-and-paper method for elucidating halide double perovskite band structures. Chemical Science 2019, 10, 11041–11053. 10.1039/C9SC03219C. 32190254 PMC7066864

[ref98] Canali C.; Jacoboni C.; Nava F.; Ottaviani G.; Alberigi-Quaranta A. Electron drift velocity in silicon. Phys. Rev. B 1975, 12, 2265 10.1103/PhysRevB.12.2265.

[ref99] Norton P.; Braggins T.; Levinstein H. Impurity and lattice scattering parameters as determined from Hall and mobility analysis in n-type silicon. Phys. Rev. B 1973, 8, 5632–5653. 10.1103/PhysRevB.8.5632.

[ref100] Kao Y. C.; Eknoyan O. Electron and hole carrier mobilities for liquid phase epitaxially grown GaP in the temperature range 200–550 K. J. Appl. Phys. 1983, 54, 2468–2471. 10.1063/1.332362.

[ref101] Casey H. C.; Ermanis F.; Wolfstirn K. B. Variation of electrical properties with Zn concentration in GaP. J. Appl. Phys. 1969, 40, 2945–2958. 10.1063/1.1658106.

[ref102] Bretschneider S. A.; Ivanov I.; Wang H. I.; Miyata K.; Zhu X.; Bonn M. Quantifying Polaron Formation and Charge Carrier Cooling in Lead-Iodide Perovskites. Adv. Mater. 2018, 30, 1707312 10.1002/adma.201707312. 29847699

[ref103] Burgos-Caminal A.; Moreno-Naranjo J. M.; Willauer A. R.; Paraecattil A. A.; Ajdarzadeh A.; Moser J. E. Hot Carrier Mobility Dynamics Unravel Competing Subpicosecond Processes in Lead Halide Perovskites. J. Phys. Chem. C 2021, 125, 98–106. 10.1021/acs.jpcc.0c08492.

