# Supplementary material for: Charge-Carrier Mobility and Localization in Semiconducting Cu2AgBiI6 for Photovoltaic Applications
Source: ACS Energy Lett. 2021 Apr 7;6(5):1729–39. doi: 10.1021/acsenergylett.1c00458 (PMC8155390; doi:10.1021/acsenergylett.1c00458)
Supplement: Supplementary file 1 — nz1c00458_si_001.pdf [file nz1c00458_si_001.pdf]

Supporting Information: Charge-carrier mobility and localization in semiconducting Cu_2AgBiI_6 for photovoltaic applications

Leonardo R. V. Buizza,[†] Adam D. Wright,[†] Giulia Longo,[†,‡] Harry C. Sansom,[†] Chelsea Q. Xia,[†] Matthew J. Rosseinsky,[¶] Michael B. Johnston,[†] Henry J. Snaith,[†] and Laura M. Herz[*,†]

†*Department of Physics, University of Oxford, Clarendon Laboratory, Parks Road, Oxford, OX1 3PU, United Kingdom*

‡*Department of Mathematics, Physics and Electrical Engineering, University of Northumbria, Ellison Place, Newcastle-Upon-Tyne, NE1 8ST, United Kingdom*

¶*Department of Chemistry, University of Liverpool, Crown Street, Liverpool, L69 7ZD, United Kingdom*

E-mail: laura.herz@physics.ox.ac.uk

Contents

1 Sample Fabrication **S-3**

2 Experimental Methods **S-4**

3 X-Ray Diffraction **S-5**

4 Fitting of Optical Absorption Spectra Using Elliott's Theory **S-8**

5 Photoluminescence and Time-Correlated Single-Photon Counting **S-10**

6 THz Photoconductivity **S-13**

Drude Factor Calculation S-13

Fitting of Temperature-Dependent Decays S-15

7 Instrument Response of OPTP Spectroscopy **S-19**

References **S-21**

1 Sample Fabrication

1.03 mmol of AgI, 1.25 mmol of CuI and 0.877 mmol of BiI_3 were dissolved in 2 ml of a mixture of DMSO and DMF with a 3:1 volume ratio. The BiI_3 was synthesised by H. Sansom, in order to avoid impurities found in commercially available BiI_3.[S1] This non-stoichiometric composition was used as it has experimentally been observed (through X-ray diffraction measurements) to be the only one leading to thin films with the expected composition.[S2] The solution was stirred at 150 °C for 30 minutes. The hot solution was filtered with a PTFE syringe filter with 0.22 μm pore size. The filtered solution was then kept at 75 °C during the entire spin-coating process. The solution was spin-coated on z-cut quartz substrates in a nitrogen-filled drybox, with a speed of 4000 rpm for 60 seconds with 4000 $rpms^{-1}$ acceleration. The spin-coated films were then annealed at 50 °C in air for 45 minutes, and, after this, at 150 °C for 3 minutes.

2 Experimental Methods

UV-Visible Absorption: UV-Visible absorption spectra were taken using a Bruker Vertex 80v Fourier-Transform Infrared (FTIR) spectrometer fitted with a transmission/reflection accessory, with a tungsten halogen source and a silicon diode detector. The temperature-dependent measurements were carried out using liquid helium, with the sample mounted in a gas exchange cryostat (Oxford Instruments, OptistatCF2), and heated with a resistive heater via a temperature controller (Oxford Instruments, MercuryiTC).

X-Ray Diffraction: X-Ray Diffraction patterns were measured using a Panalytical X'Pert powder diffractometer, using radiation from a $Cu{-}K_{\alpha 1}$ source, across 2θ values ranging from $5 - 45°$. TopasAcademic (Version 6) was used to perform Pawley fitting. In order to correct against sample tilt, the z-cut quartz peak at $2\theta = 16.43°$ was used as a reference to pin the spectra.

Time-Correlated Single-Photon Counting: Photoluminescence (PL) spectra were measured following excitation by a 398-nm pulsed laser (Picoquant, LDH-D-C-405M). The emitted PL was collected and coupled into a grating spectrometer (Princeton Instruments SP-2558), after which light was detected by a photon-counting detector (PDM series from MPD). The time-integrated PL spectra were measured at a repetition rate of 10 MHz, integrating the total counts at each wavelength over 100 ns. For the time-resolved decays a repetition rate of 1MHz was used. Timing was controlled electronically using a PicoHarp300 event timer. PL decays were measured at the high- and low-energy peak wavelengths, over the ranges $1.59 - 1.71$ eV and $1.26 - 1.31$ eV respectively. The temperature-dependent measurements were carried out using liquid helium, with the sample mounted in a gas exchange cryostat (Oxford Instruments, OptistatCF2), and heated with a resistive heater via a temperature controller (Oxford Instruments, MercuryiTC).

Optical Pump Terahertz Probe Spectroscopy: An amplified laser system (Spectra Physics, MaiTai - Empower - Spitfire) with a central wavelength 800 nm, 35-fs pulse duration and 5 kHz repetition rate was used to generate THz radiation via the inverse spin hall effect[S3] and

was detected using free-space electro-optic sampling with a 1 mm-thick ZnTe (110) crystal, a Wollaston prism and a pair of balanced photodiodes. The THz pulse was measured in transmission geometry. The pump beam was frequency-doubled to 400 nm by a β-barium-borate (BBO) crystal.

The temperature-dependent measurements were carried out using liquid helium, with samples mounted in a cold-finger cryostat (Oxford Instruments, MicrostatHe). The temperature was regulated via a temperature controller (Oxford Instruments, MercuryiTC) connected to a resistive heater and temperature sensors mounted both on the cryostat heat exchanger and at the end of the sample holder. The samples were deposited on 2 mm z-cut quartz substrates, which have excellent thermal conductivity and transparency over the THz bandwidth used in this study.[S4]

3 X-Ray Diffraction

To confirm the composition and structure of the specific samples under investigation here, X-ray diffraction (XRD) measurements were carried out on thin films of Cu_2AgBiI_6 on z-cut quartz substrates, giving the diffraction intensities shown in Figure S1. A Pawley fit was performed on the XRD patterns of the individual films used for the spectroscopic measurements. All the peaks in the XRD patterns could be fitted to a mixture of two rhombohedral phases each with a trigonal unit cell and space group $R\overline{3}m$, or to the z-cut quartz peaks of the substrate. This is consistent with the phases found by X-ray diffraction and transmission electron microscopy energy dispersive X-ray (TEM EDX) analysis in films processed using the same method by Sansom et al.[S2] In that original study, TEM EDX showed that the phases were a mixture of the targeted quaternary phase with a non-stoichiometric composition close to Cu_2AgBiI_6, and an impurity ternary phase with a non-stoichiometric composition close to Cu_2BiI_5.[S2,S5] The peak at 25° is mainly attributable to the Cu_2AgBiI_6 film, whilst the peaks at 12.8° , 29.3° and 41.5° are a combination of reflections from the Cu_2AgBiI_6 as well as

some small amount of impurities, likely to be Cu_2BiI_5. These assignments are in agreement with the original investigation of Cu_2AgBiI_6,[S2] and the above-mentioned impurities have also been examined in the original study and previous work.[S2,S5] It is worth noting that the original study of Cu_2AgBiI_6 solved the crystal structure at 100 K,[S2] raising the possibility of a phase transition arising below this temperature, as our measurements here and below were carried out to temperatures as low as 4 K. However, there are no clear discontinuities or sudden changes in behaviour of the absorption and PL spectra or in the charge-carrier dynamics below 100 K, making it unlikely that a structural phase transition has occurred as the temperature is varied.

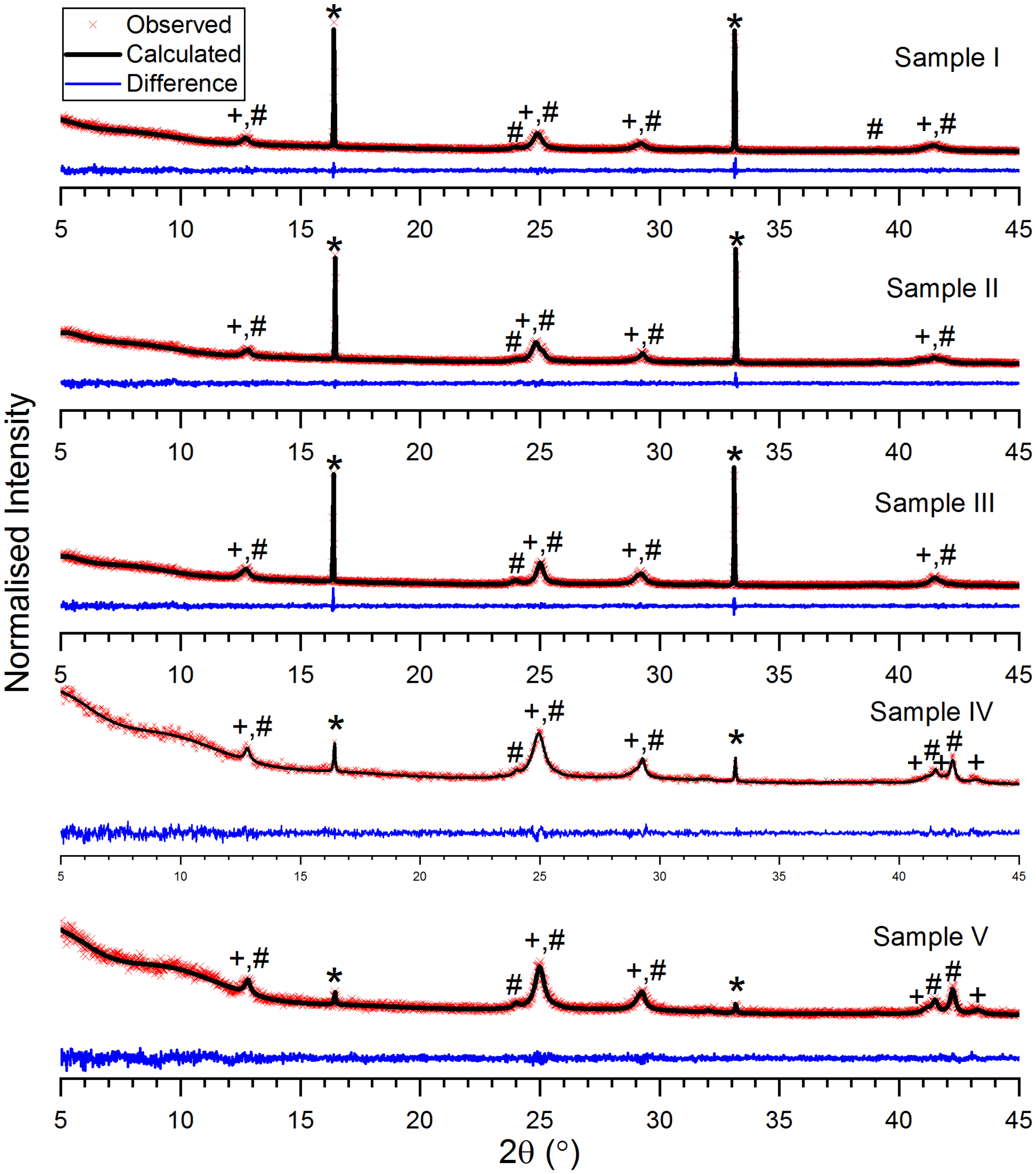

Figure S1: X-ray diffraction measurements of the Cu_2AgBiI_6 thin films that were used for the various spectroscopic experiments (red crosses), measured using a Panalytical X'Pert powder diffractometer using the Cu−Kα_1 line as incident radiation. Pawley fits are the thick black line, with the difference between the data and fits below in blue. The peaks highlighted with (+) are due to Cu_2AgBiI_6, whereas those highlighted with (#) are due to Cu_2BiI_5 impurities. The peaks highlighted with (*) at 16.3° and 33.2° are due to the quartz substrate.

4 Fitting of Optical Absorption Spectra Using Elliott's Theory

Contributions to the optical absorption of semiconductors arise from bound excitons and electron-hole continuum states. Elliott's theory can be used to describe the energy (E) dependent absorption coefficient $\alpha(E, E_{\mathrm{G}}, E_{\mathrm{B}})$ arising from a semiconductor with a band gap E_{G} and with excitons with a binding energy E_{B}, and this has previously been applied to other metal-halide perovskite systems.[S6,S7] The overall absorption coefficient can thus be described as:

$$\alpha(E, E_{\mathrm{G}}, E_{\mathrm{B}}) = \frac{b_0}{E}\sum_{n=1}^{\infty}\frac{4\pi E_{\mathrm{B}}^{3/2}}{n^3}\delta\left(E - \left[E_{\mathrm{G}} - \frac{E_{\mathrm{B}}}{n^2}\right]\right) + \frac{b_0}{E}\left[\frac{2\pi\sqrt{\frac{E_{\mathrm{B}}}{E-E_{\mathrm{G}}}}}{1-\exp\left(-2\pi\sqrt{\frac{E_{\mathrm{B}}}{E-E_{\mathrm{G}}}}\right)}\right] c_0^{-1}\mathrm{JDoS}(E) \tag{1}$$

where the joint density of states is given by $\mathrm{JDoS}(E) = c_0\sqrt{E - E_g}$ for $E > E_{\mathrm{G}}$ and 0 otherwise, and the joint density of states constant $c_0 = \frac{1}{(2\pi)^2}\left(\frac{2\mu}{\hbar^2}\right)^{\frac{3}{2}} \times 2$, where μ is the reduced effective mass of the electron-hole system. The first term in equation 1 contains the contribution from bound excitonic states, while the second term contains the contributions from the continuum states. Broadening due to electron-phonon interactions, local fluctuations in the stoichiometry of the material, and energetic disorder is represented by convolution of $\alpha(E)$ with a normal distribution $\mathcal{N}(0, \Gamma^2)$ which has a mean 0 and standard deviation Γ.

The final Elliott function $f_{\mathrm{Elliott}} = \alpha(E, E_{\mathrm{G}}, E_{\mathrm{B}}) * \mathcal{N}(0, \Gamma^2)$, where $*$ denotes convolution, was used to fit the optical absorption onset using a least-squares method, giving the fits shown in Figure S2.

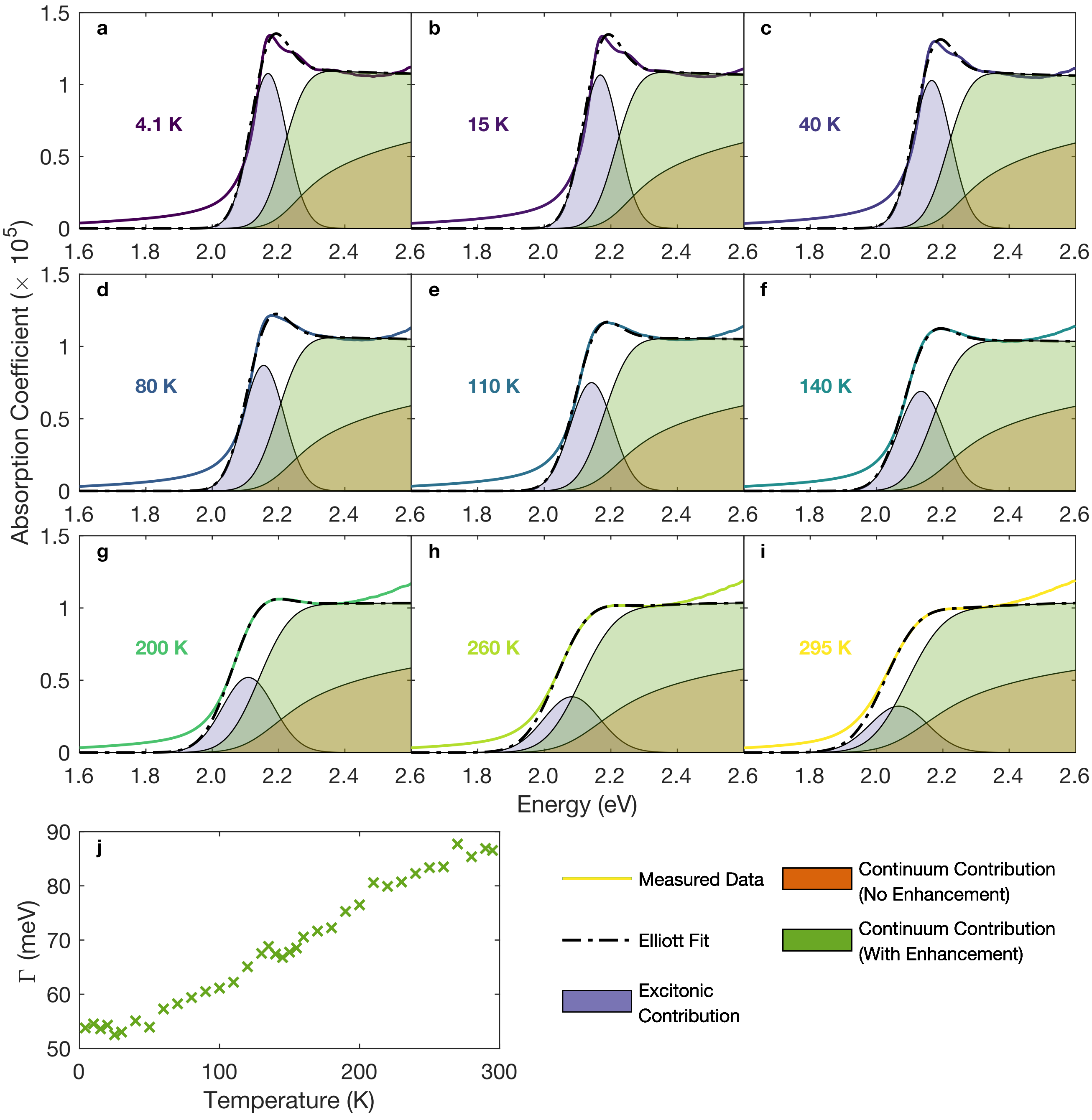

Figure S2: (a) - (i) Fits (dashed black lines) to the steady-state optical absorption spectra (solid coloured lines), across the temperature range 4 – 295 K using Elliott's theory.[S8] The excitonic (blue) and free carrier contributions, without (orange) and with (green) Coulombic enhancement are shown as the filled areas. (j) Broadening parameter Γ obtained from the Elliott fits.

5 Photoluminescence and Time-Correlated Single-Photon Counting

As mentioned in the main text, an additional infrared PL emission emerges at lower temperatures, dominating the emission spectra below 120 K (see Figure S3 (a)). This emission is between 1.26 – 1.35 eV, and blue-shifts and narrows slightly as the temperature is lowered. The PL decays for this lower-energy peak were also measured using TCSPC at a fluence of 200 $nJcm^{-2}$, with the decays shown in S3 (b). At room temperature the decays are very heterogeneous, but as the temperature is lowered they become homogeneous and the lifetime is much longer. The fits to the low-energy PL decays also give lifetimes that increase by several orders of magnitude as the temperature decreases, as has been found for emission from self-trapped states in $Rb_4AgBiBr_9$.[S9,S10,S11] However, we cannot rule out this increase in lifetime simply arising due to the shutting off of non-radiative decay pathways, especially due to the significant disorder present in Cu_2AgBiI_6.

With regard to the fitting of the PL decays, both the high- and low-energy decays were fitted with stretched-exponential fits of the form $I = I_0 e^{-(\frac{t}{\tau_{\mathrm{char}}})^{\beta}}$, and the values of τ_{av} were calculated as $\tau_{\mathrm{av}} = \frac{\tau_{\mathrm{char}}}{\beta}\Gamma(\frac{1}{\beta})$, where Γ is the gamma function.[S12] The values in table S1 marked with '*' were extracted using a monoexponential fit of the form $I = I_0 e^{-\frac{t}{\tau_{\mathrm{av}}}}$. In order to obtain the recombination rate k_1 used in the two-level model (discussed below), the rate was calculated as $k_1 = 1/(2\tau_{\mathrm{av}})$, following the convention of Richter et al.[S13] Given that the TCSPC measurements were carried out at different temperatures to the early-time optical-pump terahertz-probe (OPTP) measurements, linear interpolation between the calculated values for k_1 was used in order to obtain representative values at the required temperatures, as shown in Figure S3 (d).

Table S1: Values of parameters extracted from the fits to the PL decays. Values of τ_{av} and β are shown for the stretched-exponential fits, and the values marked with '*' are from the monoexponential fits.

**Temp (K)**	**High-Energy τ_{av} (ns)**	**High-Energy β**	**Low-Energy τ_{av} (ns)**	**Low-Energy β**
4	4100	0.24	7000$*$	N/A
40	164	0.53	7000$*$	N/A
80	145	0.45	1800$*$	N/A
120	90	0.37	1260	0.39
160	33	0.28	97	0.36
200	26	0.18	25	0.18
250	17	0.19	57	0.17
295	33	0.18	373	0.15

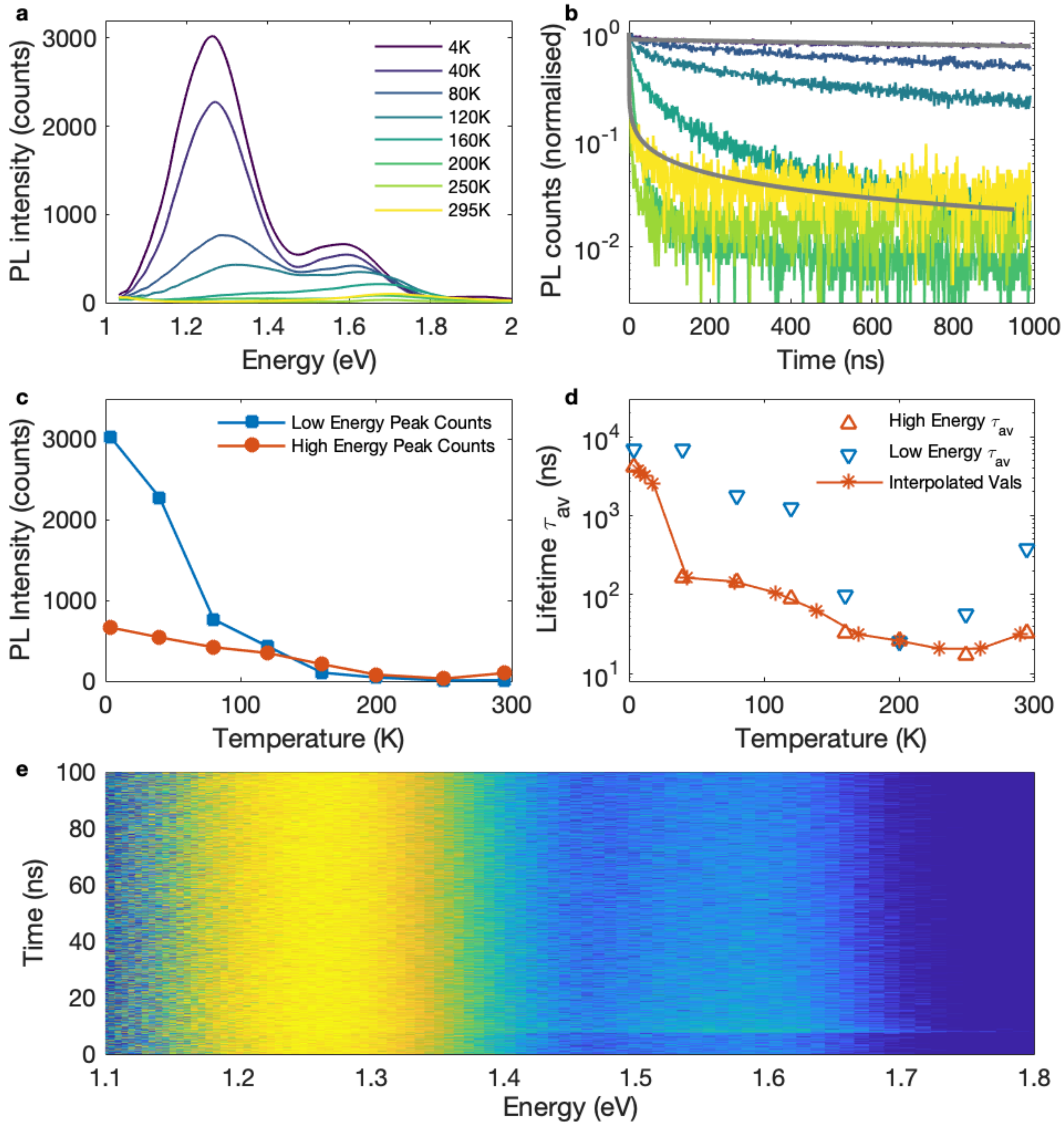

Figure S3: (a) Un-normalised photoluminescence spectra between 4 – 295 K. As the temperature is lowered, a peak in the near infra-red emerges at around 1.35 eV, and below 120 K this peak has a higher intensity than the high-energy emission. (b) Time-resolved PL decays of the low-energy emission between 1.26 – 1.31 eV, measured between 4 – 295 K. The solid grey lines at 4 K and 295 K are fits to the PL decays. (c) Intensity of photoluminescence peaks at high (1.59 – 1.71 eV) and low (1.26 – 1.31 eV) energy, plotted at each measured temperature. (d) PL lifetimes of high and low energy peaks, extracted from fits to TCSPC measurements, as discussed in the main text and above (shown as empty triangles). The stars joined by lines show the linearly-interpolated values used to obtain k_1 as an input to the two-level mobility model. (e) Time-resolved PL spectrum of Cu_2AgBiI_6 at 4 K over 100 ns, measured at an excitation fluence of 200 nJcm^{-2}, showing both high- and low-energy peaks present throughout the measurement window.

6 THz Photoconductivity

Drude Factor Calculation

The Drude model for the photoconductivity of a electrons in a solid yields a flat, zero-valued imaginary part of the photoconductivity in the THz regime.[S14–S16] In order to quantify deviations from this model, a 'Drude Factor' can be calculated as:[S17]

$$f_{\text{Drude}} = \frac{1}{x}\sum_{1}^{x} \frac{\sqrt{\text{Re}\left(\Delta T/T\right)^2}}{\sqrt{\text{Re}\left(\Delta T/T\right)^2 + \text{Im}\left(\Delta T/T\right)^2}}. \tag{2}$$

Here $\Delta T/T = \frac{T_{\text{On}} - T_{\text{Off}}}{T_{\text{Off}}}$ is the change in THz transmission at each frequency point, and x is the total number of points (in this case, we calculated f_{Drude} between $0.4 - 2.5$ THz). In the case of ideal Drude-like conductivity, $f_{\text{Drude}} = 1$, and deviations indicate other contributions e.g. from localized charge-carrier states.[S15]

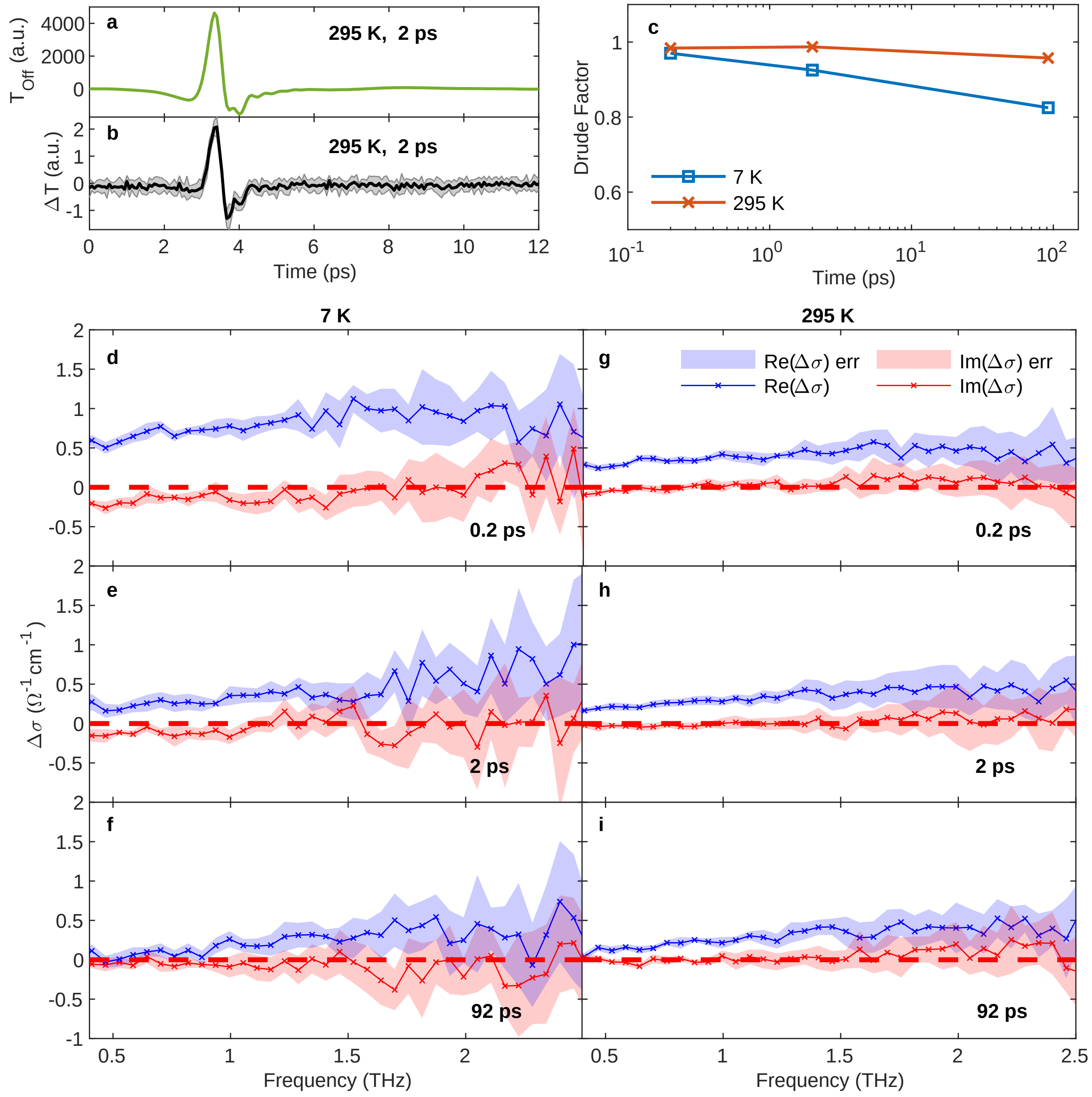

Figure S4: (a) Transmitted THz field amplitude with no photoexcitation T_{Off} for the photoconductivity spectrum measured at 295 K and a delay of 2 ps. (b) Differential THz field amplitude $\Delta T = T_{\mathrm{On}} - T_{\mathrm{Off}}$ for the photoconductivity spectrum measured at 295 K and a delay of 2 ps. (c) Calculated Drude Factor (see above for details) for each of the photoconductivity spectra shown in (d-i). Terahertz photoconductivity spectra measured on a thin film of Cu_2AgBiI_6 at delays of 0.2, 2, 92 ps at 7 K (d-f) and 295 K (g-i). Spectra were measured at a fluence of 10.2 μJcm^{-2}

Fitting of Temperature-Dependent Decays

This section discusses the temperature-dependent OPTP data shown in Main Figure 2(c) and the model used to fit this data, outlined schematically in Main Figure 2(d) and Figure S5. In general, a three-level system that includes bi- and tri-molecular recombination processes must be solved numerically, but in our case we make several simplifying assumptions in order to fit analytical solutions to our data. We do not include any 'delocalization' rate that would allow charges to go from n_{loc} back to n_{deloc}. We also assume there is no recombination from the delocalized carrier state down to the ground state: we do not observe any photoluminescence at higher energies than the PL peak at 1.59 – 1.71 eV, and given that a substantial fraction of the decay occurs within a few ps, the delocalized state depopulates so quickly that this contribution would be negligible. Further, given that our temperature-dependent OPTP measurements were carried out at the low excitation fluence of $15.3\,\mu\mathrm{Jcm}^{-2}$, we assume that the rate of charge-carrier recombination from the localized state back to the ground state is limited by monomolecular recombination, with no higher-order contributions. The recombination rate k_1 is calculated from the fits to the high-energy time-resolved PL decays, as discussed in the main text and above. Finally, we assume that all photoexcited carriers begin in the free state, such that $n_{\mathrm{deloc}}(t = 0) = n_0$ and $n_{\mathrm{loc}}(t = 0) = 0$. Note that, as discussed in the main text, we do not identify the precise nature of the localized or self-trapped state (e.g. a self-trapped hole or exciton, or a small polaron), and so the schematic is simply representative of the states and the parameters associated with them, and should not be interpreted as a band structure or energy-level diagram.

The resulting system can be schematically represented as shown in Figure S5, and the simplified model outlined can thus be solved using a set of coupled rate equations:

$$\frac{\partial n_{\mathrm{deloc}}}{\partial t} = -k_{\mathrm{loc}} n_{\mathrm{deloc}} \tag{3}$$

$$\frac{\partial n_{\text{loc}}}{\partial t} = -k_1 n_{\text{loc}} + k_{\text{loc}} n_{\text{deloc}}. \tag{4}$$

These are straightforward to solve analytically, with the solutions given by:

$$n_{\text{deloc}}(t) = n_0 e^{-k_{\text{loc}} t} \tag{5}$$

$$n_{\text{loc}}(t) = n_0 \frac{k_{\text{loc}}}{k_{\text{loc}} - k_1} \left(e^{-k_1 t} - e^{-k_{\text{loc}} t} \right). \tag{6}$$

These solutions provide us with a charge-carrier volume density, which must be converted into a sheet photoconductivity, which can then be fitted to our measured $\Delta T/T$ traces.

For the simple case of Drude-like charge-carrier transport, the photoconductivity of a material with photoexcited charge-carriers is given simply by:

$$\Delta\sigma = nq\mu, \tag{7}$$

where n is the charge-carrier volume density, q is the relevant charge, and μ is the charge-carrier mobility. In our case, where we have contributions from two different states, our overall photoconductivity is given by:

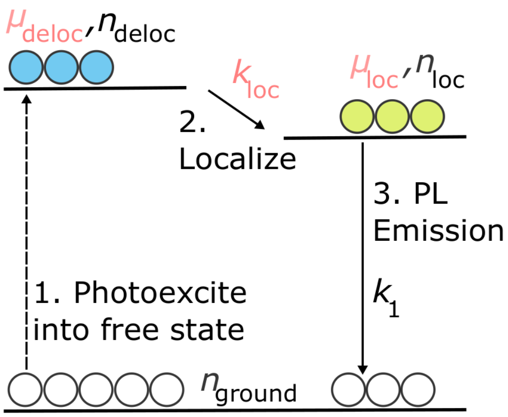

Figure S5: Schematic of the two-level mobility model used to fit the OPTP decays shown in Main Figure 2. The dotted line indicates the initial photoexcitation of charges, in our case due to pulsed laser excitation.

$$\Delta\sigma = e\left(n_{\mathrm{deloc}}\mu_{\mathrm{deloc}} + n_{\mathrm{loc}}\mu_{\mathrm{loc}}\right). \tag{8}$$

For a thin film on a thick quartz substrate, we satisfy the conditions of $d_{\mathrm{film}} << \lambda_{\mathrm{THz}} < d_{\mathrm{substrate}}$, allowing us to use the following approximation for the sheet photoconductivity of a thin film between two media of refractive indeces n_A and n_B:[S15]

$$\Delta\sigma = -\frac{\epsilon_0 c(n_A + n_B)}{d_{\mathrm{film}}}\left(\frac{\Delta T}{T}\right), \tag{9}$$

where $n_A = 1$ for vacuum and $n_B = 2.13$ for a z-cut quartz substrate.

In order to calculate our initial number of photoexcited charge-carriers, the following was used:

$$N = \frac{E\lambda}{hc}\left(1 - R_{\mathrm{pump}}(\lambda)\right)\left(1 - T_{\mathrm{pump}}(\lambda)\right), \tag{10}$$

where E is the energy contained in an optical excitation pulse of wavelength $\lambda = 400$ nm, and R_{pump} and T_{pump} are the sample reflectivity and transmittance at 400 nm. In order to convert this into a charge-carrier density per unit volume, we accounted for the effective overlap area of the optical pump and THz probe beams and the thickness of the sample, such that:

$$n_0 = \frac{N}{d_{\mathrm{film}}A_{\mathrm{Eff}}} = 1.59 \text{ x } 10^{18}\,\mathrm{cm}^{-3}. \tag{11}$$

Thus, the overall $\Delta T/T$ signal can be described using the following analytical form:

$$\frac{\Delta T}{T} = \frac{-n_0 e d}{\epsilon_0 c\left(n_A + n_B\right)}\left(\left(\mu_{\mathrm{deloc}} - \frac{\mu_{\mathrm{loc}}k_{\mathrm{loc}}}{k_{\mathrm{loc}} - k_1}\right)e^{-k_{\mathrm{loc}}t} + \frac{\mu_{\mathrm{loc}}k_{\mathrm{loc}}}{k_{\mathrm{loc}} - k_1}e^{-k_1 t}\right). \tag{12}$$

In order to account for the Instrument Response Function of the OPTP system used here (see below), the expression in Equation 12 was convolved with a normalised Gaussian pulse with $\sigma_{\mathrm{IRF}} = 0.29\,\mathrm{ps}$. Accounting for an offset in time t_0, the overall expression used to fit

the temperature-dependent OPTP data was thus:

$$\frac{\Delta T}{T}(t-t_0) = \frac{-en_0 d_{\text{film}}}{2\epsilon_0 c(n_A+n_B)}\left\{\left(\mu_{\text{deloc}} - \frac{\mu_{\text{loc}}k_{\text{loc}}}{k_{\text{loc}}-k_1}\right) e^{-k_{\text{loc}}(t-t_0)+\frac{k_{\text{loc}}^2\sigma_{\text{IRF}}^2}{2}}\,\text{Erfc}\left(\frac{k_{\text{loc}}\sigma_{\text{IRF}}^2-(t-t_0)}{\sqrt{2}\sigma_{\text{IRF}}}\right) + \frac{\mu_{\text{loc}}k_{\text{loc}}}{k_{\text{loc}}-k_1} e^{-k_1(t-t_0)+\frac{k_1^2\sigma^2}{2}}\,\text{Erfc}\left(\frac{k_1\sigma_{\text{IRF}}^2-(t-t_0)}{\sqrt{2}\sigma_{\text{IRF}}}\right)\right\}. \quad (13)$$

Here Erfc(x) is the complementary error function, defined as:

$$\text{Erfc}(x) = \frac{2}{\sqrt{\pi}}\int_x^{\infty} e^{-t^2}dt \quad (14)$$

7 Instrument Response of OPTP Spectroscopy

As a measure of the Instrument Response Function (IRF) of our THz OPTP spectroscopy systems, very finely-spaced measurements of the photoconductivity onset in silicon were carried out. This data is then fitted with an error function, via an equation of the form:

$$\mathrm{F(x)} = A\left(1 - \mathrm{Erf}(B(x - C))\right) + D \tag{15}$$

where A, B, C and D are fitting constants and the error function is defined as:

$$\mathrm{Erf}(x) = \frac{2}{\sqrt{\pi}} \int_0^x e^{-t^2} dt. \tag{16}$$

This is then converted into a Gaussian to simulate the IRF of our experimental system:

$$\mathrm{G}(x) = \alpha e^{-\left(\frac{(x-\beta)}{\sqrt{2}\sigma_{\mathrm{IRF}}}\right)^2} \tag{17}$$

where the constant σ_{IRF} is related to the fitted value B by $\sigma_{\mathrm{IRF}} = 1/\left(\sqrt{2}B\right)$, and α is simply the amplitude.

This gives the IRF shown in SI Figure S6, with $\sigma_{\mathrm{IRF}} = 290\,\mathrm{fs}$, much faster than the timescales for the ultrafast decays that we measure in the thin films of Cu_2AgBiI_6, namely the rates shown in Main Figure 3 (b).

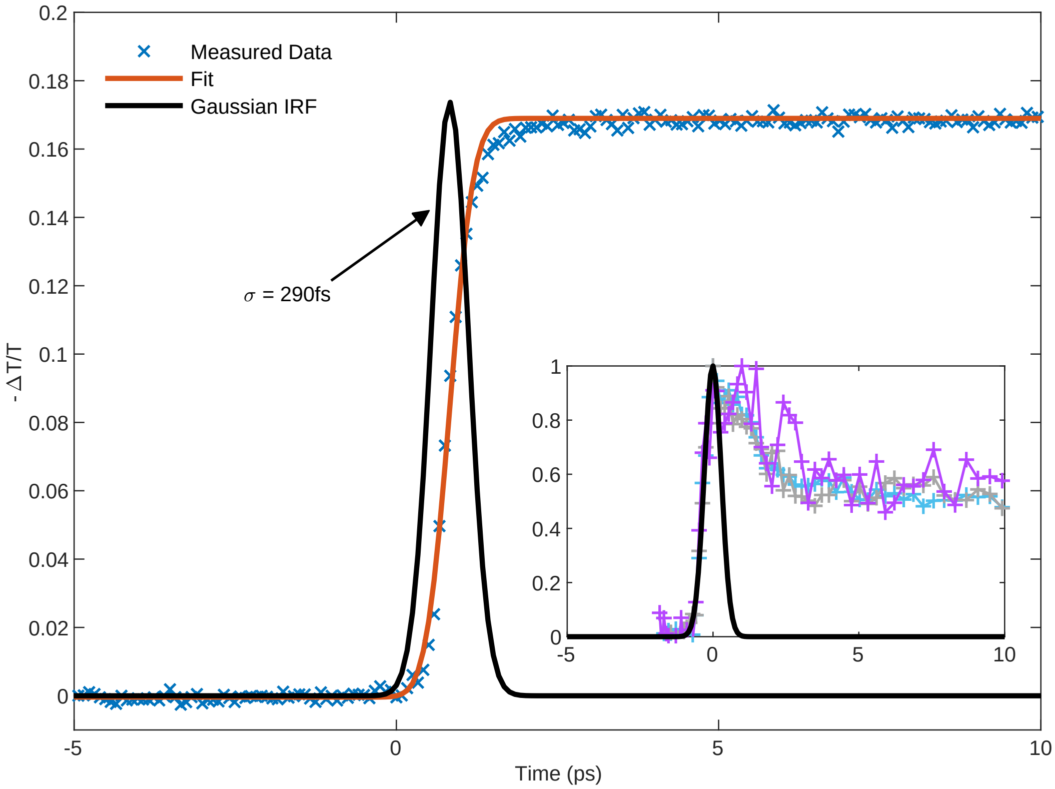

Figure S6: OPTP spectroscopy measurements on a 2 mm-thick wafer of high-resistivity silicon, pumped at 800 nm at a fluence of 14.7 µJcm^{-2}. The data is fitted with an error function in order to extract an estimate of the instrument response function of our OPTP system. The resulting Gaussian IRF has a spread of 290 fs, much shorter than the decays observed in the thin films of Cu_2AgBiI_6. For reference, the inset shows the IRF superimposed on the data shown in Figure 2 (a) of the main text, with all of the data normalised for ease of comparison.

References

(S1) Sansom, H. C.; Whitehead, G. F.; Dyer, M. S.; Zanella, M.; Manning, T. D.; Pitcher, M. J.; Whittles, T. J.; Dhanak, V. R.; Alaria, J.; Claridge, J. B.; Rosseinsky, M. J. $AgBiI_4$ as a Lead-Free Solar Absorber with Potential Application in Photovoltaics. *Chemistry of Materials* **2017**, *29*, 1538–1549.

(S2) Sansom, H. C.; Longo, G.; Wright, A. D.; Buizza, L. R. V.; Zanella, M.; Gibbon, J. T.; Abda-Jalebi, M.; Pitcher, M. J.; Dyer, M. S.; Manning, T. D.; Dhanak, V. R.; Friend, R. H.; Herz, L. M.; Snaith, H. J.; Claridge, J. B.; Rosseinsky, M. J. Highly Absorbing Lead-Free Semiconductor Cu_2AgBiI_6 for Photovoltaic Applications from the Quaternary $CuI-AgI-BiI_3$ Phase Space. *Journal of the American Chemical Society* **2021**, *143*, 3983–3992.

(S3) Seifert, T.; Jaiswal, S.; Martens, U.; Hannegan, J.; Braun, L.; Maldonado, P.; Freimuth, F.; Kronenberg, A.; Henrizi, J.; Radu, I.; Beaurepaire, E.; Mokrousov, Y.; Oppeneer, P. M.; Jourdan, M.; Jakob, G.; Turchinovich, D.; Hayden, L. M.; Wolf, M.; Münzenberg, M.; Kläui, M.; Kampfrath, T. Efficient metallic spintronic emitters of ultrabroadband terahertz radiation. *Nature Photonics* **2016**, *10*, 483–488.

(S4) Davies, C. L.; Patel, J. B.; Xia, C. Q.; Herz, L. M.; Johnston, M. B. Temperature-Dependent Refractive Index of Quartz at Terahertz Frequencies. *Journal of Infrared, Millimeter, and Terahertz Waves* **2018**, *39*, 1236–1248.

(S5) Fourcroy, P. H.; Carré, D.; Thévet, F.; Rivet, J. Structure du tétraiodure de cuivre(I) et de bismuth(III), CuBiI4. *Acta Crystallographica Section C Crystal Structure Communications* **1991**, *47*, 2023–2025.

(S6) Davies, C. L.; Filip, M. R.; Patel, J. B.; Crothers, T. W.; Verdi, C.; Wright, A. D.; Milot, R. L.; Giustino, F.; Johnston, M. B.; Herz, L. M. Bimolecular recombination

in methylammonium lead triiodide perovskite is an inverse absorption process. *Nature Communications* **2018**, *9*, 293.

(S7) Wright, A. D.; Volonakis, G.; Borchert, J.; Davies, C. L.; Giustino, F.; Johnston, M. B.; Herz, L. M. Intrinsic quantum confinement in formamidinium lead triiodide perovskite. *Nature Materials* **2020**, *19*, 1201 – 1206.

(S8) Elliott, R. J. Intensity of optical absorption by excitons. *Physical Review* **1957**, *108*.

(S9) Benin, B. M.; Dirin, D. N.; Morad, V.; Wörle, M.; Yakunin, S.; Rainò, G.; Nazarenko, O.; Fischer, M.; Infante, I.; Kovalenko, M. V. Highly emissive self-trapped excitons in fully inorganic zero-dimensional tin halides. *Angewandte Chemie International Edition* **2018**, *57*, 11329–11333.

(S10) Song, K. S.; Williams, R. T. *Self-Trapped Excitons*; Springer Berlin Heidelberg, 1993; p 404.

(S11) Fischbach, J. U.; Fröhlich, D.; Kabler, M. N. Recombination luminescence lifetimes and the self-trapped excition in alkali halides. *Journal of Luminescence* **1973**, *6*, 29–43.

(S12) Johnston, D. C. Stretched exponential relaxation arising from a continuous sum of exponential decays. *Physical Review B* **2006**, *74*, 184430.

(S13) Richter, J. M.; Abdi-Jalebi, M.; Sadhanala, A.; Tabachnyk, M.; Rivett, J. P.; Pazos-Outón, L. M.; Gödel, K. C.; Price, M.; Deschler, F.; Friend, R. H. Enhancing photoluminescence yields in lead halide perovskites by photon recycling and light out-coupling. *Nature Communications* **2016**, *7*, 13941.

(S14) Yu, P. Y.; Cardona, M. *Fundamentals of semiconductors : physics and materials properties*, 4th ed.; Springer: Heidelberg Dordrecht London New York, 2010.

(S15) Joyce, H. J.; Baig, S. A.; Parkinson, P. A review of the electrical properties of semiconductor nanowires: insights gained from terahertz conductivity spectroscopy The influence of surfaces on the transient terahertz conductivity and electron mobility of GaAs nanowires. *Semicond. Sci. Technol* **2016**, *31*, 103003.

(S16) Ulatowski, A. M.; Herz, L. M.; Johnston, M. B. Terahertz Conductivity Analysis for Highly Doped Thin-Film Semiconductors. *Journal of Infrared, Millimeter, and Terahertz Waves* **2020**, *41*, 1431–1449.

(S17) Milot, R. L.; Eperon, G. E.; Snaith, H. J.; Johnston, M. B.; Herz, L. M. Temperature-dependent charge-carrier dynamics in $CH_3NH_3PbI_3$ perovskite thin films. *Advanced Functional Materials* **2015**, *25*, 6218–6227.
